# Polydopamine-mediated EGCG-modified polystyrene microspheres for the synergistic removal of inflammatory cytokines TNF-α and bilirubin in liver failure

**DOI:** 10.1093/rb/rbag069

**Published:** 2026-04-10

**Authors:** Yunzheng Du, Zhuang Liu, Yamin Chai, Biao Wang, Lichun Wang, Zimeng Wang, Xiaofang Guo, Guanlun Zhou, Jingxuan Yang, Chunling Zhu, Leilei Yang, Xinyao Lv, Lailiang Ou

**Affiliations:** Key Laboratory of Bioactive Materials, Ministry of Education, College of Life Sciences, Nankai University, Tianjin 300071, China; Key Laboratory of Bioactive Materials, Ministry of Education, College of Life Sciences, Nankai University, Tianjin 300071, China; Key Laboratory of Bioactive Materials, Ministry of Education, College of Life Sciences, Nankai University, Tianjin 300071, China; General Hospital Tianjin Medical University, Tianjin 300052, China; Key Laboratory of Bioactive Materials, Ministry of Education, College of Life Sciences, Nankai University, Tianjin 300071, China; Key Laboratory of Bioactive Materials, Ministry of Education, College of Life Sciences, Nankai University, Tianjin 300071, China; Hubei Key Laboratory of Multi-media Pollution Cooperative Control in Yangtze Basin, School of Environmental Science & Engineering, Huazhong University of Science and Technology (HUST), Hubei, 430074, China; Key Laboratory of Bioactive Materials, Ministry of Education, College of Life Sciences, Nankai University, Tianjin 300071, China; Key Laboratory of Bioactive Materials, Ministry of Education, College of Life Sciences, Nankai University, Tianjin 300071, China; Key Laboratory of Bioactive Materials, Ministry of Education, College of Life Sciences, Nankai University, Tianjin 300071, China; Key Laboratory of Bioactive Materials, Ministry of Education, College of Life Sciences, Nankai University, Tianjin 300071, China; Key Laboratory of Bioactive Materials, Ministry of Education, College of Life Sciences, Nankai University, Tianjin 300071, China; Key Laboratory of Bioactive Materials, Ministry of Education, College of Life Sciences, Nankai University, Tianjin 300071, China; Key Laboratory of Bioactive Materials, Ministry of Education, College of Life Sciences, Nankai University, Tianjin 300071, China; Key Laboratory of Bioactive Materials, Ministry of Education, College of Life Sciences, Nankai University, Tianjin 300071, China

**Keywords:** hemoperfusion, bilirubin, tumor necrosis factor-α, epigallocatechin gallate

## Abstract

Hemoperfusion has emerged as a crucial treatment for liver failure in clinics. However, the challenge remains in the incapacity to effectively and concurrently eliminate multiple harmful toxins, including inflammatory cytokines and bilirubin. This study employed molecular docking for directing the design of adsorbents. To develop a multi-target adsorbent material for liver failure, capable of absorbing both bilirubin and tumor necrosis factor-α (TNF-α), a functional platform was constructed. This platform employs a TiO_2_-modified polystyrene-based microsphere PSVT, which was designed for bilirubin adsorption, and coated with polydopamine and grafted with epigallocatechin gallate (EGCG) for TNF-α adsorption. The results confirm that EGCG possesses a high binding affinity for TNF-α, and the introduction of EGCG as a functional moiety markedly improves the clearance effectiveness of TNF-α. Meanwhile, PSVT served as the foundation to guarantee the effective removal of bilirubin. PSVT/P/EGCG exhibits outstanding clearance performance, with a maximum adsorption capacity of 263 ng/g for TNF-α and 23.91 mg/g for bilirubin, demonstrating its high efficiency in removing both inflammatory mediators and toxins. EGCG contributes exceptional antioxidant capabilities to PSVT/P/EGCG. The adsorbent exhibits remarkable biocompatibility and stability. The multi-target adsorbent material developed in this study has an extensive spectrum of potential applications in liver failure treatment employing an artificial liver.

## Graphical abstract

**Graphical Abstract:**
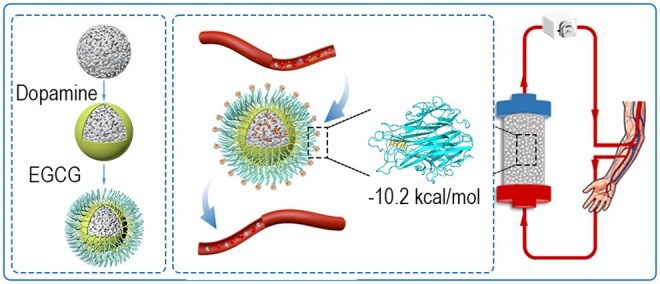

## Introduction

The liver, recognized as one of the most critical organs in the human body, is responsible for various complex physiological functions involving synthesis, metabolism, detoxification, and biotransformation. However, liver failure may develop when the liver has substantial injury from agents such as viruses or hepatic toxins, resulting in extensive hepatocyte necrosis and a significant disruption of normal physiological functions. This pathological state is characterized by a spectrum of clinical syndromes, including hyperbilirubinemia, hepatic encephalopathy, coagulopathy, and ascites [1, 2]. Approximately two million individuals die annually due to liver failure [3]. Bilirubin, a key biomarker of liver function [4], is routinely generated from aged red blood cells, combining with albumin in the circulation and then transferred to the liver for uptake [5], followed by metabolism and excretion by hepatocytes [6, 7]. Under normal conditions, the total bilirubin concentration in the blood does not exceed 17.1 μmol/L [8]. However, impaired liver function disrupts bilirubin metabolism and impacts its excretion [9]. The accumulation of excessive bilirubin in the bloodstream may result in hyperbilirubinemia, which exhibits significant hepatotoxicity and neurotoxicity, breaks the blood-brain barrier, and generates irreparable brain damage [10, 11] and, in the worst case, leads to death [12, 13].

Excessive bilirubin can disrupt the immune homeostasis of hepatocytes, causing the upregulation of pro-inflammatory cytokines and ultimately triggering widespread apoptosis and necrosis of liver cells [14]. Bilirubin can readily cross the blood-brain barrier, induce neuroinflammation, and significantly influence the progression of hepatic encephalopathy [15]. Multiple cytokines, including tumor necrosis factor-α (TNF-α), IL-6, and IL-8, are implicated in these pathological processes [16]. One of the most important inflammatory cytokines is TNF-α, an immunoregulatory cytokine released by activated macrophages [17]. In inflammatory tissues, TNF-α acts as one of the most prevalent early inflammatory mediators and a major trigger for the inflammatory cascade [18, 19]. It has been proven that high TNF-α levels cause hepatocyte necrosis and apoptosis, which are known to be the primary contributors to hepatocyte damage [16, 20]. Moreover, TNF-α could further stimulate the development of other inflammatory mediators via NF-κB signaling, thereby aggravating immune-mediated liver injury and potentially causing systemic inflammatory response syndrome [21]. Clinically, increasing blood TNF-α levels have been proven to be significantly associated with increased mortality in patients with liver failure [20].

When liver injury occurs, the accumulating bilirubin not only damages hepatocytes, resulting in cell necrosis, but also stimulates the upregulation of TNF-α synthesis and secretion [22]. In consequence, higher TNF-α levels further exacerbate hepatocellular injury, hence worsening bilirubin excretion and producing a self-perpetuating vicious cycle. This cycle ultimately culminates in extensive hepatocyte necrosis and progressive liver failure. Concurrently, the heightened inflammatory response amplifies bilirubin-induced brain damage, posing a grave threat to patient survival [23–25].

Orthotopic liver transplantation remains the definitive treatment for liver failure [26, 27]. However, its implementation is hindered by the limited availability of donor livers, the risk of postoperative immunological rejection, and the substantial financial burden involved with the surgery [28]. Prior to finding a suitable donor, an artificial liver support system can act as a temporary substitute for hepatic detoxification, bridging the gap by eliminating harmful substances from the patient’s bloodstream and extending survival time [29]. This method provides an appropriate choice in clinical practice.

Hemoperfusion, an artificial liver technique that eliminates pathogenic factors via adsorbents, has been clinically verified as an effective treatment for liver failure [30, 31]. To date, a range of adsorbent materials targeting bilirubin and cytokines have been developed [32, 33]. The initially discovered hemoperfusion adsorbent material employed for bilirubin elimination was activated carbon [34]. However, its biocompatibility is restricted, and worries over the potential leakage risk limit its clinical applicability. Ion exchange resins have also been designed for bilirubin removal; yet, they are connected with difficulties such as poor biocompatibility and the possibility of disturbing electrolyte balance and altering blood pH [35].

In recent years, various innovative materials have been studied, showing promising outcomes [36–38]. However, these materials still demonstrate drawbacks, including unsatisfactory adsorption effectiveness, limited mechanical strength, and insufficient blood compatibility. On the other hand, TNF-α exists as a trimer in the blood with a molecular weight of about 51 kDa [39]. Its large molecular size impedes efficient interaction with most adsorbents, leading to their inadequate capacity to adsorb TNF-α, which is insufficient to meet clinical requirements. Consequently, there remains a pressing requirement for the development of adsorbents capable of simultaneously targeting bilirubin in liver failure and pro-inflammatory cytokine TNF-α.

For decades, nonionic polystyrene-divinylbenzene (PS-DVB) materials have attracted considerable attention due to their prominent hydrophobicity, well-developed pore structures, and outstanding blood compatibility, rendering them exceptional adsorbents for bilirubin and cytokines removal [40–42]. Notably, the majority of commercially available adsorbent materials are primarily derived from polystyrene (PS) derivatives, including BR350 (Asahi Medical, Osaka, Japan) [43], utilized for bilirubin removal, and Cytosorb (CytoSorbents Corporation, Monmouth Junction, NJ, USA) [44], designed for cytokine adsorption. Nevertheless, none of them can accomplish the concurrent and effective elimination of both bilirubin and TNF-α. This has imposed constraints on their application in the field of liver failure. Our previous research experimentally demonstrated that the integration of TiO_2_ into the PS adsorbent enhanced both the pore structure and the mechanical strength of the material [31]. As a result, the adsorption capacity for bilirubin was significantly improved. Conventional PS-based adsorbents often demonstrate limited adsorption ability for TNF-α. The Cytosorb adsorbent, released in Europe in 2011, exploits hydrophobic interactions to remove several cytokines and is based on a PS matrix [44]. Nonetheless, TNF-α is primarily adsorbed on the material’s surface owing to its large molecular weight, resulting in inadequate adsorption efficiency, with an adsorption rate of roughly 20–30% [45]. While modifying the material with TNF-α-targeting antibodies could significantly improve the clearance efficiency, practical limitations such as poor stability, high production costs, and potential immunogenic risks prevent its wider clinical implementation [46, 47]. In this context, the development of adsorbent materials capable of concurrently and efficiently eliminating excessive bilirubin and the important pro-inflammatory cytokine TNF-α in patients with liver failure remains an enormous challenge.

Epigallocatechin gallate (EGCG) is the major polyphenolic substance in green tea and contains abundant catechol groups, conferring EGCG considerable antioxidant and anti-inflammatory activities as well as great biocompatibility [48]. Studies have indicated that one of the mechanisms by which EGCG exerts its anti-inflammatory properties is through the reduction of TNF-α activity [49, 50]. EGCG has protective benefits against TNF-α-mediated lung inflammation in mice and efficiently inhibits the formation of reactive oxygen species (ROS) [51]. Furthermore, research findings indicate that EGCG affects the NF-κB signaling pathway by targeting the connection between TNF-α and the TNF-α receptor. Specifically, EGCG binds directly to TNF-α and TNF-α receptor with an equal affinity, thereby reducing TNF-α-induced cytotoxicity [52].

**Figure 1:**
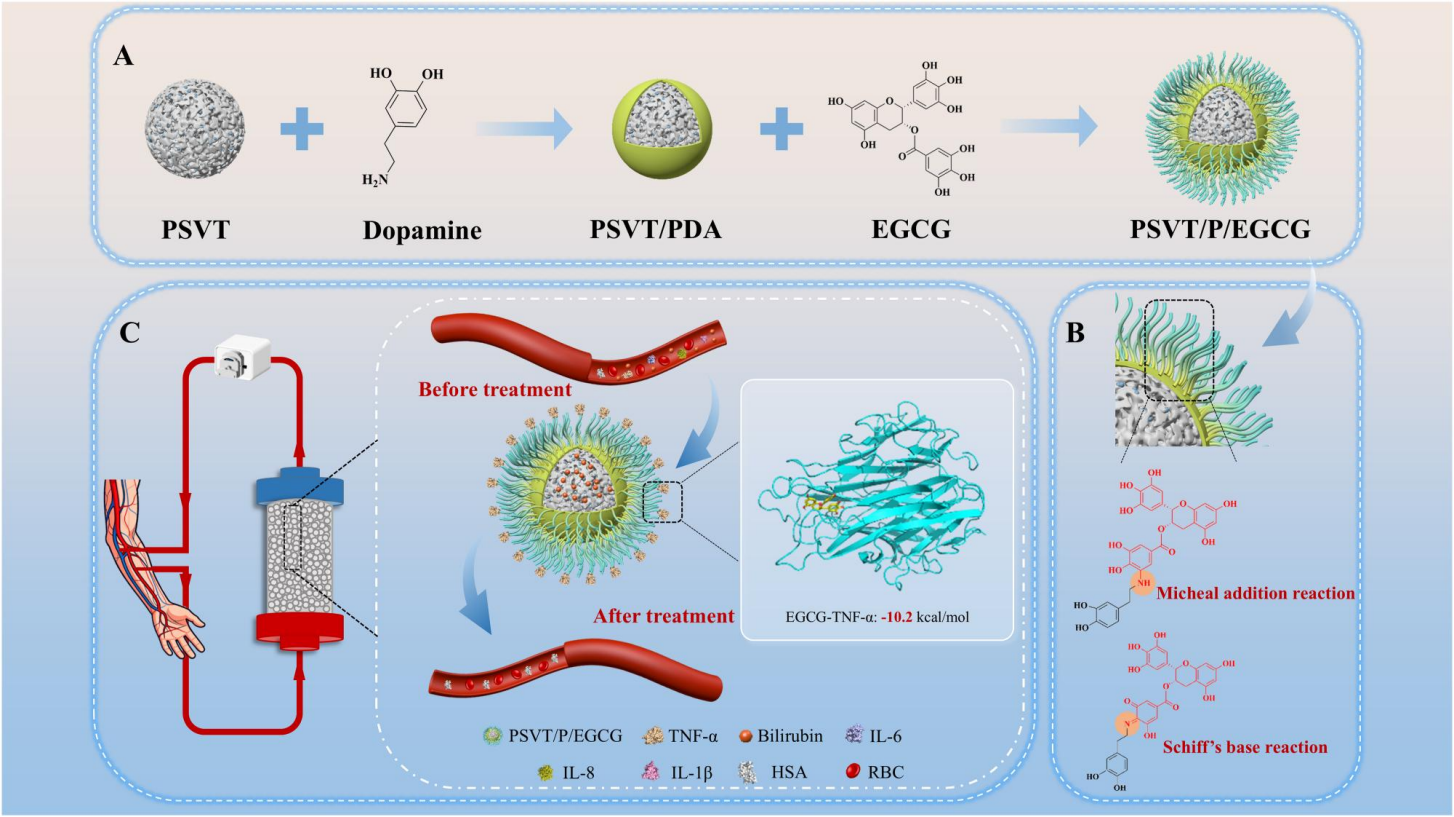
Schematic illustration of adsorbent synthesis pathways and simulated adsorption process. (**A**) and (**B**) Synthesis routes of PSVT/P/EGCG composites. (**C**) The clearance of bilirubin and TNF-α from serum components using adsorbents in the simulative hemoperfusion process.

Here, the enhanced PSVT microsphere material from our previous research was applied as the substrate. While maintaining superior bilirubin clearance efficiency, EGCG was employed as a functional ligand to improve TNF-α adsorption ability. By integrating a polydopamine (PDA)-coated PSVT, a diverse functional platform is provided. Further, in alkaline conditions, EGCG was covalently grafted onto the material surface via Michael addition and Schiff base reactions with PDA. Consequently, an adsorbent PSVT/P/EGCG was developed that efficiently removes bilirubin while simultaneously satisfying the requirement for TNF-α elimination. Additionally, this adsorbent demonstrates high adsorption ability for various cytokines, enabling liver failure patients to efficiently eliminate a significant amount of toxins by a single hemoperfusion, hence maintaining patient safety. The adsorbent also exhibits robust antioxidant properties and favorable biocompatibility. This multi-target adsorbent holds significant potential for clinical application in the treatment of patients with liver failure (Figure 1).

## Experimental section

### Materials

Recombinant human TNF-α, interleukin-1 beta (IL-1β), interleukin-8 (IL-8) and interleukin-6 (IL-6) were purchased from R&D Systems (McKinley Place, Minneapolis). Quantikine ELISA kits for human TNF-α, IL-1β, IL-8, and IL-6 were also obtained from R&D Systems. The DPPH Free Radical Scavenging Ability Assay Kit was obtained from Boxbio (Beijing, China). Bilirubin and Bovine Serum Albumin (BSA) were purchased from Sigma-Aldrich (Shanghai, China); NanoTiO_2_ and Vinyltriethoxysilane (VTES) were supplied by Aladdin (Shanghai, China). All other chemicals were of analytical grade.

### Evaluation of the binding ability of EGCG to TNF-α

#### Molecular docking simulation

The 3D structure of the TNF-α protein (PDB ID: 6OOY) was acquired from the RCSB Protein Data Bank (https://www.rcsb.org/). Crystallographic water molecules and heteroatoms were removed using PyMOL, and the revised structure was saved in PDB format. The initial structure of EGCG was obtained from the PubChem database and underwent energy minimization with the MMFF94 force field via Chem3D. Before molecular docking, both the TNF-α protein and the EGCG ligand were prepared using AutoDock Tools, including the addition of hydrogen atoms, to generate corresponding PDBQT files. A global docking strategy was implemented with a grid box of 40 Å × 50 Å × 50 Å, which was sized according to the three-dimensional structure of TNF-α protein to ensure coverage of all potential binding sites. Molecular docking simulations were conducted using AutoDock Vina, with 100 independent runs performed for each system to guarantee the reliable identification of high-confidence binding conformations. The docking results were prioritized based on their predicted binding free energies (ΔG, kcal/mol), and the highest-ranked conformation was selected for further analysis. Molecular interactions were visualized using PyMOL, while intermolecular interactions were analyzed in detail using Discovery Studio.

#### Competitive inhibition assay

The TNF-α solution with a final concentration of 1000 ng/L was co-incubated with varying concentrations of EGCG solution for 30 min. Following incubation, the concentration of TNF-α in each sample was quantified using Quantikine ELISA kits (R&D Systems, McKinley Place, Minneapolis), and the inhibition rate was calculated according to the following formula:


(1) $$\mathrm{Inhibition}\;\mathrm{ratio}\;(\%)=\frac{\left(C_{0}-C_{a}\right)}{C_{0}}*100\%,$$

where *C*_0_ (ng/L) and *C_a_* (ng/L) represent the concentrations of TNF-α when the EGCG concentration is 0 and in the experimental group, respectively.

### Synthesis of the PSVT

The synthesis of PSVT was performed following a previously established procedure. In brief, styrene (St) served as the monomer, divinylbenzene (DVB) as the crosslinking agent, VTES-modified TiO_2_ (V-TiO_2_) nanoparticles were used to increase pore structure, and toluene combined with liquid wax operated as porogens. Benzoyl peroxide (BPO) was utilized as the initiator. A homogeneous precursor mixture was prepared by combining 9 g St, 90 g DVB, 1 g V-TiO_2_, 125 g toluene, 125 g liquid wax, and 1 g BPO, followed by ultrasonication for 30 min under ice cooling to ensure uniform dispersion. The resultant mixture was rapidly added to a 1% PVA aqueous solution maintained at 45°C under mechanical stirring at 200 rpm. After 10 min, the pre-polymer generated uniform-sized droplet microspheres within the aqueous phase. The temperature was then gradually increased to 78°C, beginning the polymerization process, which was sustained for 3 h. Subsequently, the temperature was further turned to 85 and 90°C, respectively, with each stage lasting 4 h to achieve complete polymerization. Following the reaction, the material was washed with hot water to eliminate residual PVA and then put into Soxhlet extraction using petroleum ether at 90°C for 48 h to remove the porogens. Finally, the synthesized PSVT material was dried and kept for further use.

### Synthesis of the PSVT/P/EGCG

Dissolve dopamine hydrochloride in a Tris-HCl buffer to make a 1 g/L dopamine solution. Subsequently, add 1 g of PSVT material to 10 mL of the dopamine solution and incubate at 25°C for 2 h. Following this, wash the mixture three times with water and ethanol to obtain the PSVT/PDA composite. Next, combine the PSVT/PDA material with EGCG at different ratios (PSVT/PDA:EGCG = 10:1, 10:2, 10:4, 10:8, and 10:10) in a Tris-HCl solution and incubate at 37°C for predetermined durations (4, 8, 12, 24 h). Finally, wash the product three times with water to obtain the PSVT/P/EGCG material. By changing the EGCG content and reaction time, a series of PSVT/P/EGCG composites were successfully synthesized.

### Characterization of PSVT/P/EGCG

The surface and cross-sectional morphologies of the materials were observed using a field emission scanning electron microscope (FESEM, Apreo S LoVac, USA). The elemental distribution on the material surface was characterized via Energy-Dispersive X-ray Spectroscopy (EDS, Apreo S LoVac, USA). The particle size distribution was measured and analyzed with a laser diffraction particle size analyzer, followed by Gaussian fitting for further data processing. The zeta potentials of materials were determined via the Zetasizer Nano ZS90 analyzer (Malvern, UK). The pore structure was characterized using the Brunauer–Emmett–Teller (BET) method with a BELSORP miniX instrument (Japan). The pore size distribution was determined by analyzing the desorption branches of the isotherms according to the Barrett–Joyner–Halenda (BJH) method at 77 K. X-ray photoelectron spectroscopy (XPS, Thermo Fischer ESCALAB 250Xi, USA) was employed to characterize the chemical structure and surface element composition of the adsorbents. The thermogravimetric analysis (TGA, Mettler Toledo, Sweden) was used to investigate the degradation behavior of materials at a heating rate of 5°C/min from 50 to 800°C in an N_2_ atmosphere.

The total phenolic content was determined using the Folin–Ciocalteu method. In brief, 790 μL of distilled water, 50 μL of the Folin–Ciocalteu reagent and 5 mg of adsorbents were mixed for 1 min. Subsequently, 150 μL of 20% (w/v) aqueous sodium carbonate solution was added, and the resulting mixture was incubated at room temperature in the dark for 2 h. Following incubation, the absorbance was measured at 750 nm [53].

### Adsorption experiments

#### Research on adsorption in simulated serum

To investigate the adsorption ability of TNF-α and bilirubin *in vitro*, a series of adsorption experiments was carried out. First, to screen for the optimal properties of the adsorbent, adsorption studies were conducted utilizing PSVT/P/EGCG materials with different EGCG concentrations and grafting times. The adsorption capacity of the adsorbent for bilirubin was also verified. Next, the adsorption kinetics and isotherms were examined to investigate the theoretical maximum adsorption capacity and adsorption mechanism. Lastly, given that the microenvironment can considerably influence the adsorption process, it was necessary to investigate the adsorbent’s efficacy under varying albumin concentrations, ion strengths, and temperatures. Specific experimental details are available in the Supplementary Information.

#### Research on adsorption in human plasma

Additional research was conducted into the adsorbent’s adsorption capacity in a human plasma environment (supplied by Tianjin Medical University General Hospital). The adsorbent’s adsorption capacity for TNF-α and bilirubin in plasma conditions was thoroughly assessed. The human hyperbilirubinemia plasma was spiked with recombinant human TNF-α at a target concentration of 1000 ng/L. Prior to use in adsorption experiments, the beads were allowed to swell fully in 0.9% saline solution. The adsorbents were added to plasma with a ratio of adsorbent to plasma of 1:10 and shaken at 160 rpm for 2 h at 37°C. The concentrations of TNF-α were determined using Quantikine ELISA kits (R&D Systems, McKinley Place, Minneapolis). The concentrations of TBIL, DBIL, and IBIL were measured. The adsorbent’s adsorption capacity for TNF-α and other pro-inflammatory cytokines was thoroughly assessed. Notably, a mini-perfusion system was built to test the adsorbent’s efficacy in removing various toxins under real-time perfusion conditions. A 1-mL adsorption column was packed with adsorbent. Subsequently, 10 mL of human plasma containing cytokines and bilirubin was perfused through the column at 37°C in the dark, at a constant flow rate of 1 mL/min for 2 h. Two commercially available adsorbents—CYT (CytoSorb^®^, Cytosorbents Inc., USA) and BPR (BR-350, Asahi Kasei Medical Co., Ltd., Japan)—were employed as reference controls. All adsorption experiments were performed under identical conditions to the test groups.

### The experiment on the antioxidant capacity of PSVT/P/EGCG

The antioxidant capacity of the adsorbents was tested using the DPPH Free Radical Scavenging Assay Kit. According to the instruction manual, 2.5 mg of the material was added to 1 mL of the DPPH working solution and incubated at room temperature in the dark for 30 min. Subsequently, the absorbance of the supernatant was measured at 515 nm, and the DPPH radical scavenging efficiency was calculated. Trolox functioned as the positive control, whereas PBS operated as the negative control.

### Biosafety evaluation of PSVT

#### Blood compatibility of PSVT

Before the experiment, the adsorbent was incubated overnight with sterile physiological saline at room temperature to ensure complete equilibration. To evaluate the hemolytic potential of the adsorbent, whole blood was collected from the ear vein of New Zealand White rabbits using EDTA-K_2_ anticoagulant tubes. The blood was diluted with sterile 0.9% (w/v) sodium chloride solution (physiological saline) at a volume ratio of 4:5 (blood: saline), and the resulting mixture was centrifuged at 1500 × g for 5 min to pellet erythrocytes. After removal of the supernatant plasma and buffy coat, the erythrocyte pellet was resuspended in 9-mL saline to yield a standardized red blood cell suspension for hemolysis assays. Subsequently, 2 g of the adsorbent was added to 5 mL of physiological saline as the experimental group. For the control groups, 5 mL of distilled water (positive control) and 5 mL of physiological saline (negative control) were employed. Then, 100 μL of the red blood cell suspension was added to each group and incubated at 37°C for 1 h. Afterward, the specimens were centrifuged at 2500 rpm for 10 min. Finally, the absorbance of the supernatant was measured at 545 nm, and the hemolysis rate was estimated using the formula provided in previous studies [54].

The anticoagulant properties of PSVT/P/EGCG were evaluated. For the preparation of platelet-poor plasma (PPP), 4 mL of blood extracted from a rabbit heart was placed into sodium citrate anticoagulated tubes and centrifuged at 4000 rpm for 15 min. Next, 0.2 g of the adsorbent was introduced into 2 mL of PPP and maintained at 37°C for 1 h with shaking. The resulting supernatant was harvested and sent to the hospital for determination of activated partial thromboplastin time (APTT) and thrombin time (TT). Furthermore, routine blood testing was conducted. In detail, 2 mL of blood collected from a rabbit heart was transferred to EDTA·K_2_ anticoagulated tubes, followed by the addition of 0.2 g of the adsorbent. This mixture was kept at 37°C for 1 h before the blood was carefully withdrawn and analyzed using a fully automatic blood cell counter. Blood biochemical assays were performed under identical experimental conditions. Two commercially available adsorbents, CYT and BPR, were employed as reference controls for hemolysis, coagulation, and blood routine assays; all corresponding experiments were conducted under identical conditions to those applied to the experimental group. To evaluate the impact of the adsorbent on human blood cells, 0.2 g of the adsorbent was co-incubated with 2-mL blood for 2 h. Subsequently, the morphology of the blood cells was observed after staining with Wright-Giemsa reagent. The non-specific adsorption of albumin by the adsorbent was also examined. Here, 50 mg of the adsorbent was added to 5 mL of a 30 g/L BSA solution, and the mixture was shaken at 37°C for 2 h. Subsequently, the BCA assay kit was used to quantify the reduction in BSA levels.

#### Cytotoxicity experiment

To validate the cytocompatibility of PSVT, cytotoxicity tests were conducted, encompassing a CCK-8 assay and a live–dead staining assay. Specific experimental details are provided in the Supplementary Information.

### Statistical analysis

All adsorption and biosafety experiments were performed with *n* = 3 independent biological replicates. Data are presented as mean ± SD. Comparisons among multiple groups were analyzed by one-way analysis of variance followed by Tukey’s post-hoc test. A *P*-value of < 0.05 was considered statistically significant.

## Results and discussion

### Evaluation of the binding ability of EGCG to TNF-α

#### Molecular docking

**Figure 2:**
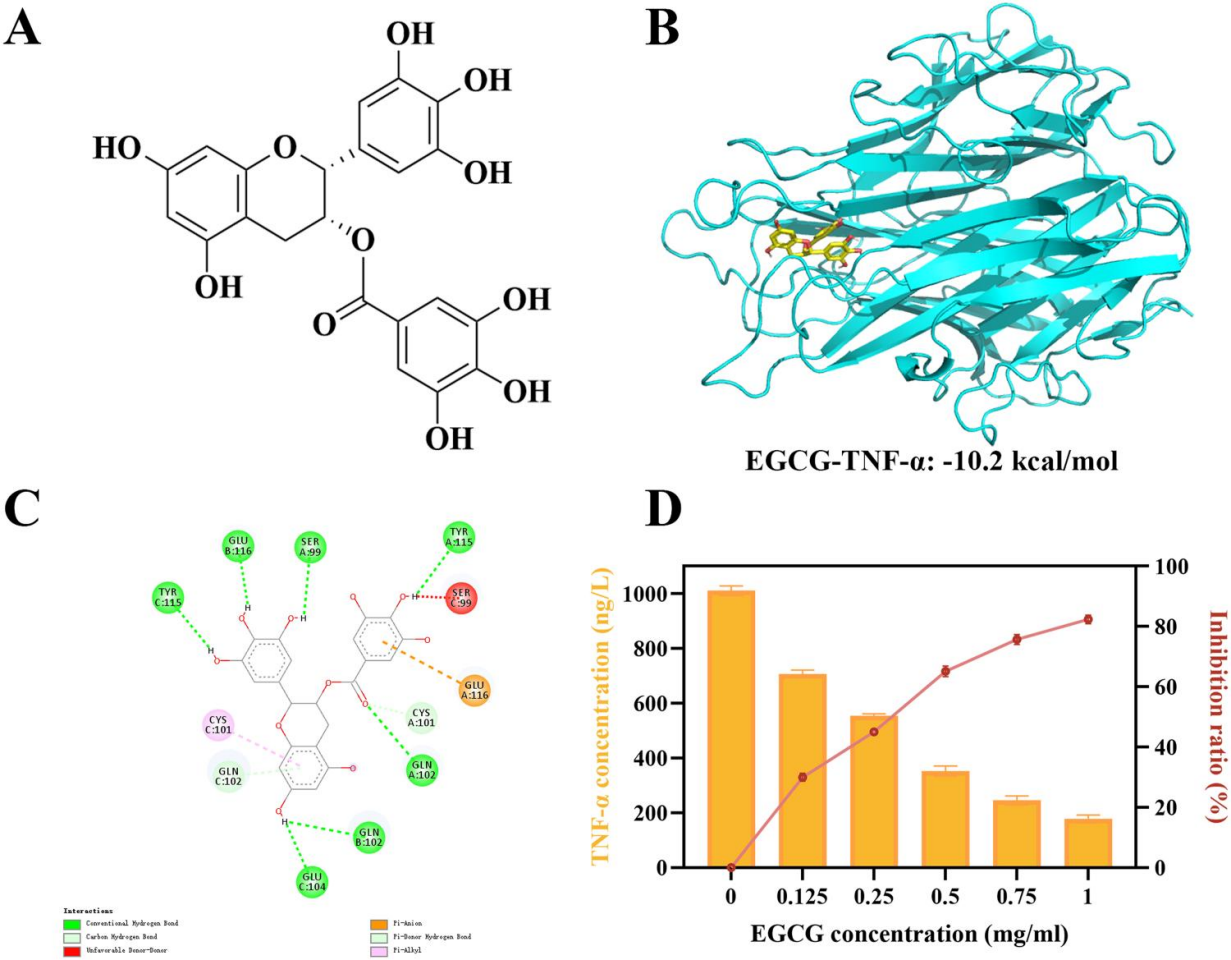
(**A**) The chemical structure of EGCG. (**B**) The molecular docking of EGCG with TNF-α. (**C**) The binding conformation of EGCG and TNF-α. (**D**) Competitive inhibition of EGCG on TNF-α.

Molecular docking is a widely utilized computational method for analyzing the interactions between target proteins and small molecules [55, 56]. In this research, molecular docking was performed to analyze the binding mode and interaction mechanisms between EGCG and TNF-α. The docking results shown in Figure 2B and Supplementary Figure S1 demonstrated the lowest binding free energy of -10.2 kcal/mol for the EGCG-TNF-α complex, which is substantially lower than the typically recognized threshold of −6 kcal/mol. This reveals a substantial binding affinity and suggests that EGCG may exhibit notable biological action in relation to TNF-α.

The binding conformation of the complex is visually illustrated in the accompanying image (Figure 2C), where TNF-α is displayed as a light-green ribbon structure, and EGCG is shown using a yellow-red stick-and-ball depiction. Interaction analysis suggests that EGCG primarily binds to the active site of TNF-α via hydrogen bonds (green dashed lines), carbon-hydrogen bonds (light-green dashed lines), π-cation interactions (orange dashed lines), and π-alkyl interactions (pink dashed lines). Key residues involved in these interactions include Ser99, Cys101, Gln102, Glu104, Tyr115, and Glu116. Notably, EGCG forms three hydrogen bonds with Gln102 and one hydrogen bond with each of the other active residues. Additionally, the benzene ring of EGCG engages in strong hydrophobic interactions with Cys101 and a π-anion interaction with Glu116.

Overall, the molecular docking studies illustrate that the binding stability between EGCG and TNF-α is mainly preserved by hydrogen bonding networks and hydrophobic interactions. These non-covalent interactions jointly contribute to the structural integrity of the polyphenol-protein complex. The findings provide a solid theoretical foundation for the development of EGCG-based functionalized adsorbent materials.

#### Competitive inhibition experiment

The competitive inhibitory experiments further established the binding between EGCG and TNF-α. As shown in Figure 2D, by co-incubating TNF-α with increasing concentrations of EGCG, a concentration-dependent increase in EGCG-TNF-α binding was detected, accompanied by a commensurate rise in the inhibition rate of TNF-α detection. This indicates that EGCG binds with TNF-α with high affinity, significantly interfering with antibody recognition of the TNF-α protein. These findings provide substantial experimental data supporting the rational design of EGCG-based adsorbent materials for targeted applications.

### Characterization of PSVT/P/EGCG

**Figure 3:**
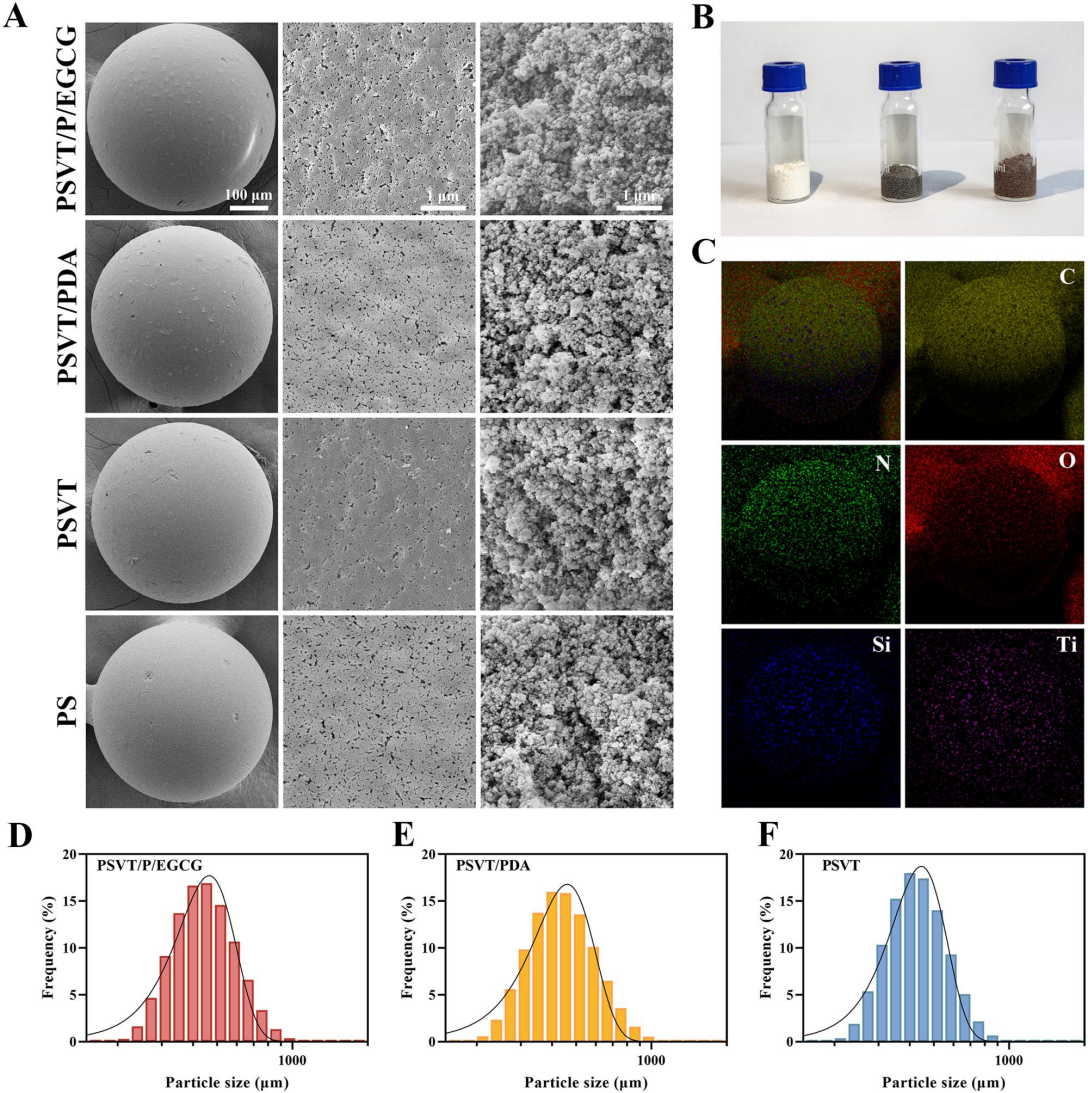
Characterization of PSVT/P/EGCG. (**A**) The morphologies of the surface pore structure of PS, PSVT, PSVT/PDA, and PSVT/P/EGCG. (**B**) Optical images of PSVT (white), PSVT/PDA (black), and PSVT/P/EGCG (light red). (**C**) EDS graphic of PSVT/P/EGCG. The size distribution of PSVT/P/EGCG (**D**), PSVT/PDA (**E**), and PSVT (**F**).

The optical images and particle size distribution of the materials are shown in Figure 3B, D, and F. The PSVT went from white to black following polydopamine coating and further changed to light red after being grafted with EGCG, which indicated that Michael addition and Schiff base reactions occurred between EGCG and PDA, which were bonded to the material surface. All materials demonstrated uniform spherical shapes with an average diameter of approximately 450 μm, as determined by Gaussian distribution fitting. The detailed particle size distribution is summarized in the Supplementary Table S1. The SEM images in Figure 3A further provided a detailed visualization of the surface and cross-sectional morphology of the materials. The modifications had a negligible impact on the fundamental surface structure and porous nature of the materials. Upon magnification, the materials were clearly observed to possess a highly interconnected porous structure, with numerous penetrating mesoporous channels visible within. Additionally, the elemental distribution of the material was examined using EDS, as shown in Figure 3C. It is worth noting that, distinct from the noisy signals of PSVT, a stronger and more uniformly distributed N element signal could be discerned in PSVT/P/EGCG and PSVT/PDA due to the PDA modification.

**Figure 4:**
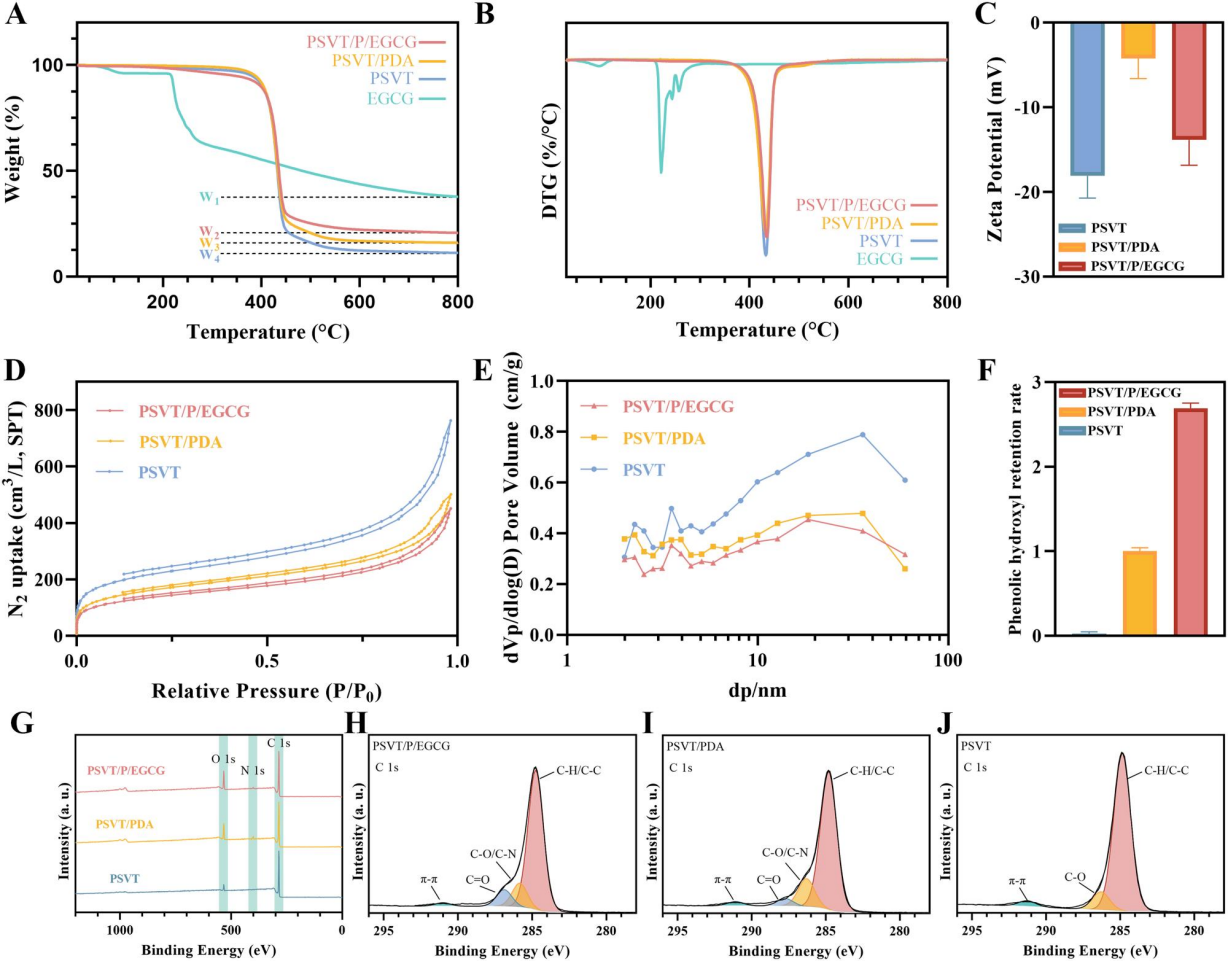
The TGA(**A**) and DTG(**B**) curves of adsorbents. (**C**) The Zeta potential picture of materials. (**D**) The BET analysis curves of materials. (**E**) PSDs by the BJH method of materials. (**F**) Total phenolic content of adsorbents. (**G**) The XPS survey spectrum and high-resolution C 1s XPS spectra (**H–J**) of three materials (mean ± SD, *n* = 3 for each group).

As shown in Figure 4A and B, the TGA analysis of the materials was performed under a nitrogen atmosphere, and DTG curves were obtained through derivation. For the EGCG, the first-stage thermal weight loss in the range of 100–200°C could be attributed to water evaporation. A more considerable mass loss occurred above 200°C, mostly due to the oxidation and thermal decomposition of EGCG at elevated temperatures, resulting in a final mass loss of 62.25 wt%. Notably, the TGA curves of the resin materials before and following grafting were comparable, with a pronounced rapid mass loss observed around 400°C, which was ascribed to the thermal degradation of the polymer matrix. However, after the grafting of PDA and EGCG, the weight loss of the material decreased, and the ultimate residual mass of the PSVT/P/EGCG material was 79.32 wt%. Based on these observations and using the following formula, the amount of EGCG grafted onto the material was calculated to be 12.41%.


(3) $$\mathrm{The}\;\mathrm{content}\;\mathrm{of}\;\mathrm{EGCG}\;(\mathrm{wt}\%)=\frac{W_{2}-W_{3}}{W_{1}}*100\%$$

where *w*_1_, *w*_2_, and *w*_3_ represent the leftover amount of EGCG, PSVT/P/EGCG, and PSVT/PDA.

The zeta potential of the material further confirms the successful preparation of the adsorbent. From Figure 4C, owing to the residual initiator and partially adsorbed oxygen free radicals on the surface of the PS material, the Zeta potential of PSVT was measured at −18.1 ± 2.6 mV. After polydopamine coating, the potential increased to −4.2 ± 2.3 mV. However, the potential was reduced to −13.8 ± 3.1 mV following EGCG grafting, which could be attributed to the negative charge of EGCG due to the presence of numerous phenolic hydroxyl groups. This result indicates the successful modification of the material with EGCG.

The Folin–Ciocalteu test was employed to quantify the changes in total phenolic content during the modification process. As demonstrated in Figure 4F, the hydroxyl content increased after PDA coating and further increased following EGCG grafting, thereby further validating the successful grafting of EGCG onto the material.

The pore structure of the material was analyzed by the BET method, and the results are displayed in Figure 4D and E and Supplementary Table S2. The grafting of polydopamine and EGCG partially obscured the pore structure, leading to a decrease in specific surface area (from 777.12 to 484.87 m^2^/g) together with reductions in total pore volume and average pore diameter. Despite theoretical concerns that structural modifications could adversely impact adsorption efficacy, experimental findings demonstrate that the incorporation of EGCG would not substantially hinder bilirubin clearance. Moreover, the presence of EGCG enhanced the adsorption of TNF-α, rendering the detrimental impact of reduced specific surface area negligible.

The surface components of the materials were examined by XPS. As depicted in Figure 4G, elements carbon (C) and oxygen (O) were detected on the surfaces of all three materials. Distinctively, in contrast to PSVT, signal peaks of element nitrogen (N) were also identified on the surfaces of PSVT/P/EGCG and PSVT/PDA. Only a weak signal corresponding to silicon (Si) was detected in PSVT, and no titanium (Ti) was detected in any of the three materials, which might be ascribed to its low content and the shielding of the detection sites subsequent to coating. The O element in PSVT can be attributed to the adsorbed oxygen on the material surface and the residual benzoyl peroxide initiator. The variations in the contents of elements C, N, and O are presented in the Supplementary Table S3. The N element demonstrates a tendency of initial increase from 0% to 2.43%, followed by a decrease to 1.30%, while the O element exhibits an upward trend. The modification of PDA introduces elements O and N, and the modification of EGCG leads to an increase in the content of the O element and a relative reduction in the content of the N element. Simultaneously, as demonstrated in Figure 4H–J, the development of the O–C=O deconvolution peak in the high-resolution C 1s element spectrum subsequent to modification could be attributed to the aldehyde groups formed during the polymerization of polydopamine. The alterations in the deconvolution peaks of the O–C=O bond and the C–N bond after EGCG grafting also substantiate the reaction between EGCG and PDA. The variations in the composition of chemical bonds in the O 1s and N 1s spectra also provide support for the success of the grafting process. Table 1 summarizes key physicochemical parameters of the adsorbents.

**Table 1:** Summary of physicochemical parameters of the adsorbents.

Adsorbents	PSVT/P/EGCG			PSVT/PDA			PSVT		
Particle size (μm)	452			445			437		
BET surface area (cm^2^/g)	484.87			575.96			777.12		
Pore volume (cm^3^/g)	0.6970			0.7754			1.1807		
EGCG loading (%)	12.41%			NA			NA		
Elemental ratios (%)	**C**	**N**	**O**	**C**	**N**	**O**	**C**	**N**	**O**
	84.78	1.30	13.93	85.84	2.43	11.73	92.26	0	7.74

### Adsorption experiments

**Figure 5:**
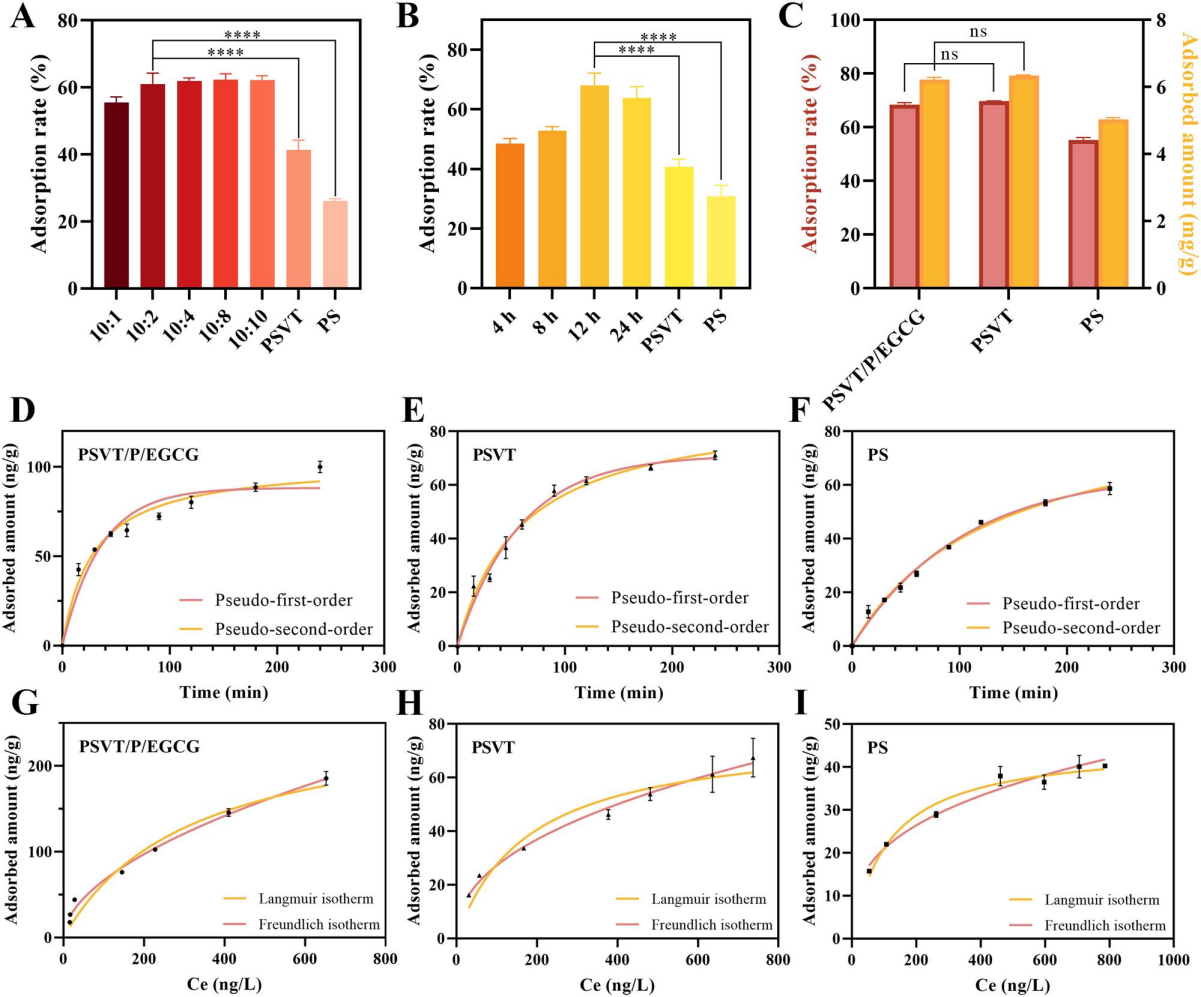
The TNF-α adsorption experiments of adsorbents with different EGCG contents (**A**) and reaction time (**B**). (**C**) The bilirubin adsorption experiments of adsorbents. (**D–F**) The adsorption kinetics curves of three materials. (**G–I**) The adsorption isotherms curves of three materials (mean ± SD, *n* = 3 for each group), ****P < 0.0001, ns.: not significant.

A series of adsorption experiments was carried out to validate the scavenging action of the adsorbent. Firstly, after the synthesis of PSVT/P/EGCG, its enhanced adsorption capacity for TNF-α was confirmed through systematic adsorption experiments. The optimal grafting conditions were explored by altering the mass of EGCG and the reaction time. As shown in Figure 5A and B, when the feeding ratio of EGCG to PSVT microspheres was 10:2, the adsorption of the material on TNF-α reached saturation. Continuously increasing the amount of EGCG did not lead to a further improvement in adsorption, while extending the grafting time enhanced the adsorption, reaching the optimum at 12 h, which might be attributed to the saturation of material contact sites and pore blockage caused by excess amounts. Under these conditions, the adsorption rate and adsorption capacity of the material for TNF-α could reach 84.7 ± 1.59% and 68.2 ± 1.09 ng/g, significantly higher than those of the PSVT material before grafting (68.8 ± 1.09% and 55.3 ± 0.91 ng/g). Meanwhile, whether the grafting of EGCG interfered with the adsorption of bilirubin was also examined. The results in Figure 5C suggested that the grafting of EGCG did not significantly interfere with the adsorption of bilirubin due to the abundant adsorption sites for bilirubin within the PSVT matrix. The adsorption rate and adsorption capacity of the PSVT/P/EGCG for bilirubin still reach 68.4 ± 0.68% and 6.2 ± 0.05 mg/g, respectively.

Adsorption kinetics can be utilized to describe the rate at which solutes are adsorbed by adsorbents. The adsorption kinetics of the material for TNF-α were investigated. From Figure 5D–F, it was revealed that the PSVT/P/EGCG material exhibited rapid adsorption properties within the initial 0–45 min. This behavior may be attributed to the abundant specific surface area of the adsorbent and the abundance of affinity binding sites. The driving force at the solid-liquid interface facilitates the rapid adsorption of TNF-α molecules onto the material, particularly under conditions of high initial concentrations. As the adsorption progresses over time, the number of available adsorption sites diminishes, leading to a gradual reduction in adsorption until equilibrium is eventually reached. Data fitting was performed using pseudo-first-order and pseudo-second-order kinetic models [57, 58]. The results revealed that for PSVT, the correlation coefficient of the pseudo-first-order kinetic model ($R_{1}^{2}$ = 0.9806) was higher than that of the pseudo-second-order kinetic model ($R_{2}^{2}$ = 0.9797). Additionally, the equilibrium adsorption capacity Q calculated using the pseudo-first-order kinetic model was 71.22, which closely matched the actual experimental value (72.97 ng/g). This indicates that the adsorption process of TNF-α on PSVT aligns more closely with pseudo-first-order kinetics, with hydrophobic interactions between the adsorbent and TNF-α serving as the predominant driving force. However, after EGCG grafting, the adsorption behavior of TNF-α on the PSVT/P/EGCG material shifted toward better fitting with the pseudo-second-order kinetic model ($R_{2}^{2}$ = 0.9676 > $R_{1}^{2}$ = 0.9278), and the equilibrium adsorption capacity calculated using the pseudo-second-order kinetic model reached 103.01, closely approximating the experimentally measured value (102.45 ng/g). This suggests that the incorporation of EGCG modifies the adsorption mechanism of TNF-α, influenced not only by hydrophobic interactions between the adsorbent carrier and TNF-α but also by the high binding affinity between EGCG and TNF-α, which is attributed to the numerous hydroxyl groups in EGCG and its rich hydrogen bonding interactions with TNF-α molecules. The pseudo-second-order kinetic model further illustrates that the adsorption process of this adsorbent is mainly driven by chemical adsorption.

The theoretical maximum adsorption capacity was obtained through the examination of adsorption isotherms. From Figure 5G–I, according to Giles’ classification of liquid adsorption isotherms, the adsorption isotherms of the three materials for TNF-α all fall into the H-2 category [59]. As the relative concentration of TNF-α increases, the adsorption capacity of the adsorbent for TNF-α molecules gradually rises. However, the slope of PSVT/P/EGCG is larger than that of PSVT and PS, which is attributed to EGCG providing more high-affinity TNF-α binding sites. The data were fitted and analyzed using the Langmuir and the Freundlich isotherm models [57, 60–62]. The results indicate that both the Langmuir and the Freundlich models demonstrate satisfactory performance in describing the adsorption process. However, the Freundlich model exhibits higher *R*^2^ values. Specifically, for PSVT/P/EGCG, the *R*^2^ value reaches 0.9880. This superior fit can be explained by the fact that the Langmuir model assumes monolayer adsorption, whereas TNF-α protein molecules exhibit interactions that lead to trimer formation [61, 62]. According to the simulation results of the Langmuir model, the theoretical maximal adsorption capacity of PSVT/P/EGCG for TNF-α could reach 263.6 ng/g, significantly surpassing that of PSVT at 77.01 ng/g. In contrast, the Freundlich model reveals that the adsorption mechanism involves multilayer adsorption, encompassing not only hydrophobic interactions but also numerous hydrogen bond interactions. Additionally, the parameter 1/*n*, which indicates the adsorption intensity, plays a critical role in evaluating the favorability of the adsorption process. When 0 < 1/*n* ≤ 1, the adsorption process is considered favorable [63]. The 1/*n* values for all three materials fall within the range of 0–1, indicating that TNF-α molecules are readily adsorbed onto the adsorbent surfaces and pores.

The adsorption kinetics and isotherms of bilirubin on the adsorbents were systematically investigated. As illustrated in Supplementary Figures S11–S14 and Tables S5 and S7, the grafting of EGCG did not significantly reduce the material’s adsorption capacity for bilirubin, nor did it modify the fundamental adsorption mechanism, which remained consistent with monolayer chemical adsorption primarily driven by hydrophobic interactions. These findings align well with our prior experimental results [31].

The microenvironment in patients with liver failure experiences significant disorders, and any alterations in the internal environment may potentially influence the adsorption efficiency. Therefore, the adsorption behavior of the adsorbent was systematically investigated under varying albumin concentrations, ionic strengths, and temperatures.

**Figure 6:**
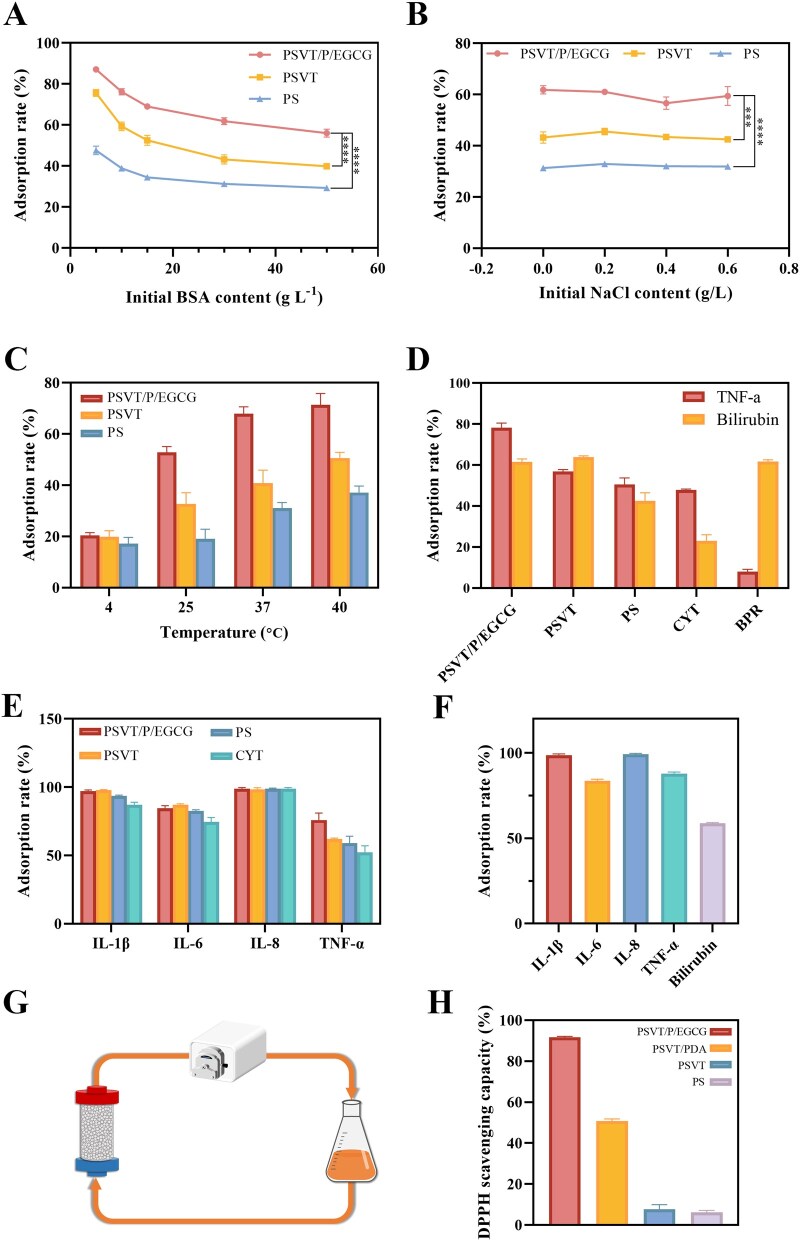
(**A**) Effect of albumin in the adsorption medium (*T* = 37°C, *t* = 2 h). (**B**) Effect of ionic strength of the solution (*C*_BSA_ = 30 g/L, *T* = 37°C, *t* = 2 h). (**C**) Effect of temperature (*C*_BSA_ = 30 g/L, *t* = 2 h). (**D**) Adsorption of bilirubin and TNF-α in the hyperbilirubinemic plasma system. (**E**) The adsorption of multiple pro-inflammatory cytokines in the plasma system. (**F**) The combined adsorption of bilirubin and multiple pro-inflammatory cytokines in the plasma system and simulation diagram of clinical perfusion micro-model (**G**). (**H**) The DPPH radical scavenging capacity experiment (mean ± SD, *n* = 3 for each group), ***P < 0.001, ****P < 0.0001.

The results in Figure 6A demonstrated that with the increase in albumin concentration, the adsorption of PSVT/P/EGCG exhibited a downward trend. Albumin competes with TNF-α for the hydrophobic adsorption sites of the adsorbent, thereby reducing the binding between the adsorbent and TNF-α. Additionally, the increase in albumin concentration also elevates the mass transfer resistance of TNF-α protein molecules. The effect of albumin concentration on bilirubin adsorption follows an inverse relationship: increasing albumin concentration leads to a progressive decline in adsorption efficiency (Supplementary Figure S16). This attenuation arises from large-volume bilirubin-albumin complexes, competing with free bilirubin for binding sites, occupying adsorption sites and sterically hindering access of free bilirubin, thereby reducing effective site availability and overall adsorption capacity. As shown in Figure 6B, the alteration of ionic strength disrupts the hydrogen bond interaction between EGCG and TNF-α, leading to a certain degree of decline in adsorption capacity, but it still remains significantly higher than that of PSVT. Attributed to the enhanced Brownian motion of TNF-α molecules at elevated temperatures, improving the chance of molecular binding between the adsorbent and TNF-α, the adsorption capacity of the adsorbent for TNF-α enhances as the temperature rises (Figure 6C). However, considering that high temperatures may contribute to protein denaturation and inactivation, and given the physiological relevance of human body temperature, 37°C was determined to be the optimal adsorption temperature. Similarly, the adsorption capacity of the adsorbents for bilirubin increased with rising temperature (Supplementary Figure S15)—mirroring the trend observed for TNF-α. Bilirubin adsorbed onto the material predominantly through hydrophobic interactions. Increasing temperature promotes adsorption via three interrelated mechanisms: (i) reduction of solution viscosity, which accelerates diffusion in the external boundary layer and intraparticle pores of bilirubin [64]; (ii) thermally driven conformational isomerization of bilirubin from the less stable cis to the extended trans configuration, which decreases steric hindrance and improves molecular accessibility to adsorption sites [65]; and (iii) negligible impact on the desorption equilibrium of hydrophobically bound bilirubin [66]. Collectively, these factors resulted in a monotonic increase in adsorption capacity with rising temperature.

The combined adsorption of bilirubin and TNF-α in the plasma system was further explored and displayed in Figure 6D. Two commercially available adsorbents, CYT (targeting cytokines) and BPR (targeting bilirubin), were selected as controls. The results indicated that while CYT effectively adsorbed TNF-α and BPR efficiently removed bilirubin, neither exhibited substantial cross-adsorption capacities for the other substance. In contrast, the PSVT/P/EGCG material demonstrated exceptional dual-adsorption ability for both bilirubin and TNF-α, achieving adsorption efficiencies of 78.17 ± 1.95% and 61.64 ± 1.16% in plasma, respectively. The plasma bilirubin clearance efficacy, stratified by bilirubin speciation, is summarized in Supplementary Table S8. As shown, the PSVT/P/EGCG group achieved effective removal of both direct and indirect bilirubin. Notably, levels of indirect and total bilirubin were reduced to clinically acceptable thresholds (total bilirubin <2.0 mg/dL; indirect bilirubin <1.5 mg/dL). In contrast, although direct bilirubin exhibited a substantial reduction relative to the baseline, its post-adsorption concentration remained marginally above the normal reference range (<0.5 mg/dL), attributable to its markedly elevated initial concentration in the plasma sample. This residual level is amenable to further reduction through extended perfusion duration or repeated treatment cycles.

Multiple pro-inflammatory cytokines collectively contribute to the inflammatory response associated with liver failure, mediating cell necrosis and directly impacting patient prognosis. Therefore, the efficient clearance of various cytokines is of critical importance. The adsorption efficiency of the PSVT/P/EGCG material on several key pro-inflammatory cytokines was further evaluated under human plasma conditions. The results shown in Figure 6E demonstrated that the PSVT/P/EGCG material exhibits exceptional adsorption capacity for multiple cytokines. Specifically, the clearance rates of IL-1β and IL-8 were nearly 100%, while those of IL-6 and TNF-α were 84.66 ± 1.41% and 75.92 ± 4.24%, respectively. Notably, except for IL-8 (where clearance approached 100% for both materials), the PSVT/P/EGCG material outperformed the commonly used commercial adsorbent CYT in clearing the other three cytokines.

Subsequently, a micro-perfusion device was constructed using a peristaltic pump and a small perfusion column to simulate the clinical hemoperfusion process in patients with liver failure. The result is shown in Figure 6F. Following a 2-h treatment, cytokines and bilirubin in the plasma were considerably and efficiently eliminated. This series of experiments substantiated that the PSVT/P/EGCG material exhibits exceptional clearance capabilities for toxic substances, including bilirubin and various pro-inflammatory cytokines, in the blood of patients with liver failure, particularly outstanding in the clearance of TNF-α protein molecules that are difficult to remove. These findings suggest that PSVT/P/EGCG has the potential to serve as an innovative and effective adsorbent material for the treatment of liver failure, effectively eliminating toxins from the patient’s body, improving vital signs, and safeguarding life safety.

Patients with liver failure exhibit substantial oxidative stress, leading to widespread damage. The polyphenolic groups in EGCG provide significant free radical scavenging and antioxidant capabilities to materials. To test the antioxidant ability of PSVT/P/EGCG, we performed the DPPH assay, utilizing a positive control defined as 100% radical clearance (not shown). Described in Figure 6H, PSVT/P/EGCG revealed a significant antioxidant activity with a clearance rate of 92.1%. PSVT/PDA also displayed moderate antioxidant capability (49.9%), attributed to the phenolic hydroxyl groups in polydopamine. In contrast, PSVT and PS demonstrated little antioxidant effects. These data suggest that EGCG considerably increases the antioxidant performance of PSVT/P/EGCG, hence presenting preventive potential against oxidative damage during hemoperfusion therapy.

### Biosafety assessment of PSVT/P/EGCG

**Figure 7:**
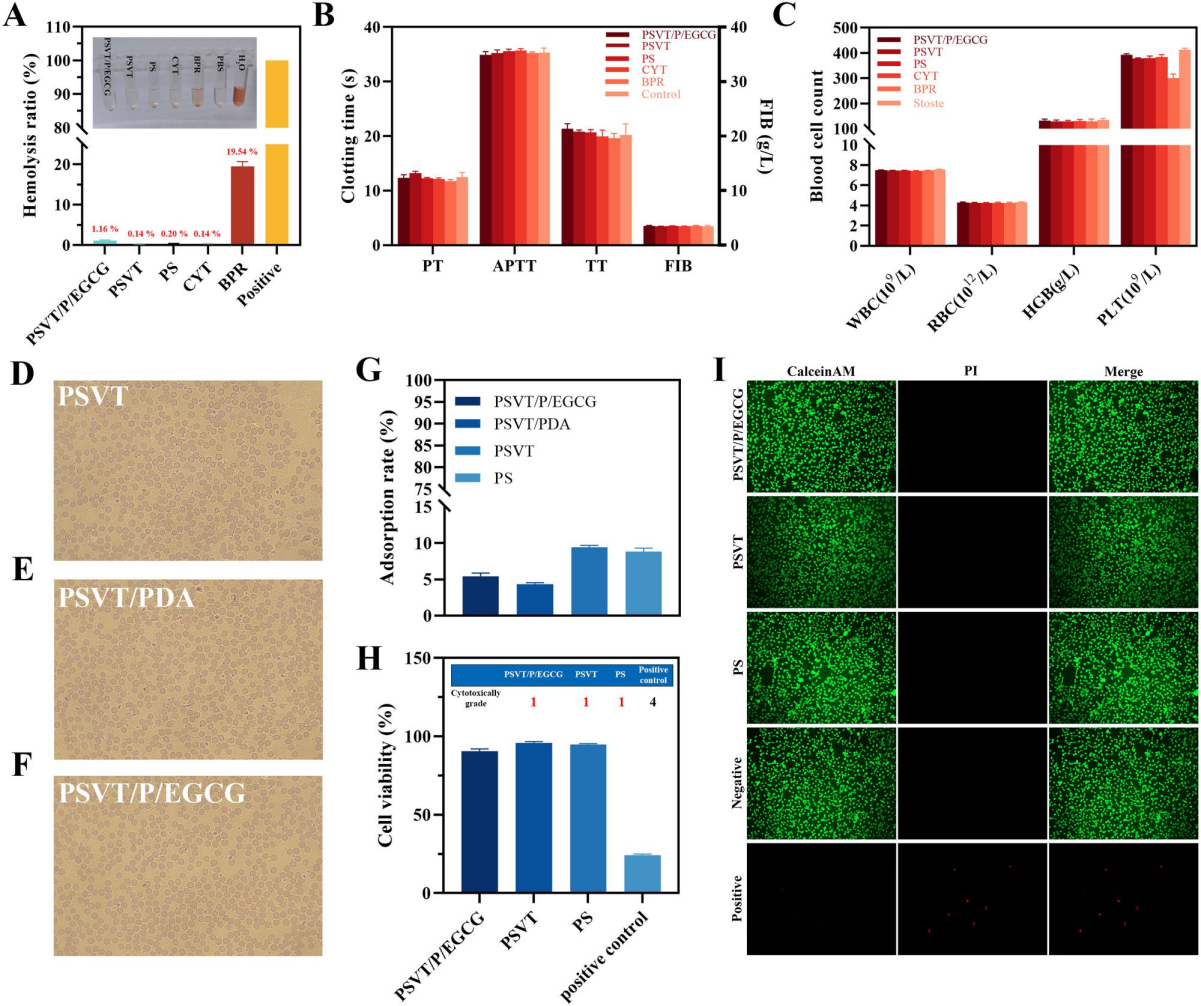
Blood compatibility and biocompatibility evaluation of the adsorbents: (**A**) hemolysis rate for the control groups and adsorbents, where the inset displays a digital image of red blood cells (RBCs) following incubation with different samples. (**B**) PT, APTT, TT, and FIB levels in platelet-free plasma after incubation with various adsorbents. (**C**) Comparison of blood cell counts between normal whole blood and blood treated with adsorbents. (**D–F**) The morphology of human RBCs after incubation with the adsorbents. (**G**) The non-specific adsorption results of BSA by adsorbents. (**H, I**) Cell viability of the HE293T cells of the adsorbents by the CCK-8 assay, where the inset shows the cytotoxicity grade (mean ± SD, *n* = 6 for each group).

Hemoperfusion adsorbent materials come into direct contact with the blood or plasma of patients during treatment. Unanticipated hemolysis and coagulation during hemoperfusion can pose a serious threat to patient safety. Therefore, the biological safety of these materials is subject to extremely stringent requirements. To ensure patient safety and minimize the risk of complications, we evaluated the blood compatibility performance of the materials through a series of experiments. The hemolysis test results in Figure 7A revealed that the hemolysis rate of PSVT/P/EGCG was only 1.16%. In contrast, the hemolysis rate of the commercially available bilirubin-specific adsorbent BPR reached 19.54%, indicating that BPR is unsuitable for whole-blood perfusion and is restricted to plasma perfusion applications. According to ASTM F756-17(2025), biomaterials exhibiting a hemolysis rate <2% are classified as non-hemolytic; those with a rate of 2–5% are categorized as exhibiting mild hemolysis; and those exceeding 5% are deemed hemolytic. These findings confirmed that PSVT/P/EGCG meets the clinical standard for hemolysis and exhibits excellent outstanding blood compatibility without inducing hemolysis. As shown in Figure 7D–F, the staining experiment of the incubated blood cells further confirmed that the red blood cells remained in a favorable condition, exhibiting intact morphologies without any symptoms of denaturation or lysis. An adsorbent material that triggers a coagulation reaction may lead to thrombus development in the blood, potentially causing severe adverse reactions such as embolism or organ dysfunction. Therefore, investigating the impact of the adsorbent on blood coagulation function is of critical importance. The APTT and TT can be used to evaluate the antithrombotic properties of *in vitro* samples, while prothrombin time (PT) assesses the exogenous coagulation ability, and fibrinogen adsorption evaluates the procoagulant activity. The results shown in Figure 7B demonstrated that, compared with the control group, the four coagulation-related parameters for the PSVT/P/EGCG material did not exhibit significant changes, indicating that PSVT/P/EGCG does not interfere with the normal blood coagulation function. Likewise, the blood biochemical assay results shown in Supplementary Table S11 indicate that PSVT/P/EGCG did not alter blood composition. The impact on whole blood was also evaluated using a blood cell counter, with the results presented in Figure 7C. The results revealed that the adsorbent did not cause significant reductions in red blood cells, white blood cells, hemoglobin, or platelets. In patients with liver failure, the albumin concentration within the body decreases, and non-specific adsorption of albumin by the adsorbent can significantly affect treatment outcomes. The albumin adsorption experiment was conducted to verify the non-specific adsorption characteristics of the material. The result shown in Figure 7G revealed that at an albumin concentration of 30 g/L, the PSVT material adsorbed approximately 9.36% of albumin due to the hydrophobic interactions between PSVT and albumin. It is worth noting that the polydopamine coating effectively reduced non-specific adsorption, decreasing albumin adsorption to 4.57%, which was attributed to the enhanced anti-fouling performance conferred by the hydrophilic characteristics of PDA [67]. Grafting EGCG slightly increased non-specific adsorption to 5.16%. This phenomenon can be attributed to two interrelated factors: first, the multi-aromatic ring scaffold of EGCG inherently increases its hydrophobic character; second, covalent grafting of EGCG consumes accessible hydrophilic phenolic hydroxyl groups. Overall, the modification process significantly decreased non-specific adsorption, consequently boosting the blood safety of the adsorbent material.

Finally, the cytotoxicity of the adsorbent was evaluated to confirm its effect on cell viability. Figure 7H indicated the relative viability of the positive control group was 24.3%, which met the evaluation criteria. The results demonstrated that the cell proliferation rate of the PSVT/P/EGCG material was 90.57%, and fluorescence staining in Figure 7I confirmed that the cells in the PSVT/P/EGCG group were in a healthy state, indistinguishable from those in the negative control group. This finding verifies the robust cytocompatibility of the PSVT/P/EGCG material. This series of experiments comprehensively demonstrated that PSVT/P/EGCG has become safe and non-toxic to blood and does not induce adverse reactions upon contact with blood. Therefore, it holds great potential as a hemoperfusion adsorbent for clinical applications in treating liver failure.

## Conclusions

This study aims to address the critical requirement for the removal of bilirubin and TNF-α during hemoperfusion in patients with liver failure. We initially utilized molecular docking simulations to examine the interaction between epigallocatechin-3-gallate and TNF-α protein molecules, demonstrating a significant binding affinity. Subsequently, EGCG was conjugated onto the surface of PSVT utilizing a polydopamine-based functional platform, leading to the successful development of an innovative composite adsorbent material, PSVT/P/EGCG, proficient in concurrently eliminating accumulated bilirubin and the pro-inflammatory cytokine TNF-α. Thorough material characterization and experimental assessments revealed that PSVT/P/EGCG has a substantial specific surface area, extensive pore structure, and superior biocompatibility. The efficacy of bilirubin elimination is particularly outstanding owing to the strong porous and hydrophobic structure of PSVT. Furthermore, the introduction of EGCG markedly improves the composite material’s affinity and capacity for the removal of TNF-α, for which the theoretical maximum adsorption capacity could reach 263.6 ng/g. Variations in the blood microenvironment demonstrated negligible impact on the adsorption efficacy, affirming the material’s stability under physiological settings. Moreover, real-time perfusion studies performed in a plasma environment confirmed the material’s exceptional clearance effectiveness for multiple toxins. The presence of EGCG further confers potent ROS scavenging capability to the adsorbent, thereby mitigating oxidative stress-induced damage in patients. The composite material demonstrated outstanding biocompatibility, exhibiting neither hemolytic nor coagulation-inducing effects and preserving the normal morphology and function of blood cells after perfusion. In summary, PSVT/P/EGCG provides a potential composite adsorbent with great efficacy in removing bilirubin and TNF-α, paired with outstanding biocompatibility. This study presents an innovative approach for designing multifunctional adsorbents aimed at various toxins in liver failure and shows great potential use in artificial liver-based blood purification systems. Future studies will focus on scaling up the synthesis and conducting *in vivo* efficacy evaluations in murine and porcine models of hyperbilirubinemia to further validate the translational potential of PSVT/P/EGCG.

## Supplementary Material

rbag069_Supplementary_Data
