# Supplementary material for: Polydopamine-mediated EGCG-modified polystyrene microspheres for the synergistic removal of inflammatory cytokines TNF-α and bilirubin in liver failure
Source: Regen Biomater. 2026 Apr 10;13:rbag069. doi: 10.1093/rb/rbag069 (PMC13135362; doi:10.1093/rb/rbag069)
Supplement: rbag069_Supplementary_Data [file rbag069_supplementary_data.docx]

**Supplementary Information**

**Polydopamine-Mediated EGCG-Modified Polystyrene Microspheres for the Synergistic Removal of Inflammatory Cytokines TNF-α** **and Bilirubin in liver failure**

*Yunzheng Du^a^, Zhuang Liu^a^, Yamin Chai^a,b^, Biao Wang^a^, Lichun Wang^a,c^, Zimeng Wang^a^, Xiaofang Guo^a^, Guanlun Zhou^a^, Yueshuang Jin^a^, Jingxuan Yang^a^, Chunling Zhu^a^, Xinyao Lv^a^, Leilei Yang^a^, and Lailiang Ou^a^**

*^a^Key Laboratory of Bioactive Materials, Ministry of Education, College of Life Sciences, Nankai University, Tianjin, 300071, China.*

*^b^General Hospital Tianjin Medical University, Tianjin, 300052, China*

*^c^School of Environmental Science and Engineering, Huazhong University of Science and Technology, Wuhan, 430074, P.R. China*

*E-mail: [ouyll@nankai.edu.cn](mailto:ouyll@nankai.edu.cn)

**Supplementary Experimental Procedures, Figures, and Tables**

**Supplementary Experimental Procedures**

**Competitive inhibition experiment**

To exclude potential interference of EGCG with TNF-α antibody binding during the ELISA detection, EGCG solutions were pre-incubated with the immobilized anti-TNF-α in a 96-well plate prior to TNF-α quantification via Quantikine ELISA kits (R&D Systems, McKinley Place, Minneapolis). Briefly, serial concentrations of EGCG solution (200 μL per well) were added to the antibody-coated wells and incubated at room temperature for 2 hours. After incubation, the supernatant was removed and the wells were thoroughly washed with wash buffer to remove unbound components. Subsequently, TNF-α solutions were added, and antigen detection was performed following the manufacturer’s instructions.

**Adsorption experiments**

Given the clinically observed hypoalbuminemia in patients with liver failure[1], all adsorption experiments were conducted using 1× phosphate-buffered saline (PBS) supplemented with bovine serum albumin (BSA) to prepare a simulated serum with a physiologically relevant total protein concentration of 30 g/L. Before the experiment, TNF-α was dissolved in simulated serum solution with a TNF-α concentration of approximately 1000 ng/L. A series of adsorption tests were performed by adding the adsorbent with the simulated serum at varied adsorption ratios. The TNF-α concentration before and after adsorption had been measured using Quantikine ELISA kits (R&D Systems, McKinley Place, Minneapolis). The configuration and detection methods of the IL-6, IL-8, and IL-1βsimulated serum solution are the same as those of TNF-α. For the bilirubin adsorption experiment, bilirubin was first dissolved in a small amount of DMSO and Na_2_CO_3_, followed by dispersion in a 30 g/L BSA solution to generate a bilirubin-simulated serum solution with a bilirubin concentration of approximately 15 mg/dL. Since bilirubin is prone to photodecomposition, the adsorption studies were conducted in the dark by adding the adsorbent with the simulated serum at varying adsorption ratios. The bilirubin concentration before and after adsorption was evaluated by diazo color development and measured at 560 nm using an ultraviolet (UV-)-vis spectrometer (UV-1800, Mapada)[2]. The adsorption rate and adsorption capacity were determined using the following equations, respectively[3].

$$\begin{aligned} Adsorption rate\left( \% \right)=\frac{C- C_{a}}{C}*100\#\left( S1 \right) \end{aligned}$$

$$\begin{aligned} Q_{e} =\frac{\left（ C-C_{a} \right）*V}{m}\#\left( S2 \right) \end{aligned}$$

Where: C is the bilirubin/TNF-α concentration before being adsorbed; Ca is the bilirubin/TNF-α concentration after being adsorbed; Qe (mg/g) is adsorption amount; V (mL) is the volume of bilirubin/TNF-α solution; and m (g) is the mass of adsorbents.

**Effect of PSVT/P/EGCG adsorbents with different EGCG** **contents and reaction time**

A wet weight of 30 mg of the adsorbents was added to 3 mL of 1000 ng/L TNF-α solution, which was shaken at 160 rpm for 2 h at 37 °C.

**Adsorption capacity of PSVT/P/EGCG on bilirubin**

A wet weight of 30 mg of the adsorbents was added to 3 mL of 15 mg/dL bilirubin solution, which was shaken at 160 rpm for 2 h at 37 °C.

**Adsorption kinetics**

One milliliters (wet volume) of adsorbents were added to 30 mL of 1000 ng/L TNF-α solutions. Then, the mixtures were shaken at 160 rpm for 4 h at 37 °C. Subsequently, 100 μL samples were extracted at a set time and quantitative analysis was performed. The kinetics data were analyzed according to the pseudo-first-order and pseudo-second-order equations[4]:

$$\begin{aligned} ln\left[ \frac{Q_{e}}{Q_{e}-Q_{t}} \right]=k_{1}t\#\left( S3 \right) \end{aligned}$$

$$\begin{aligned} Q_{t}=\frac{k_{2} Q_{e}^{2}t}{1+k_{2}Q_{e}^{2}t}\#\left( S4 \right) \end{aligned}$$

where Q_e_ and Q_t_ (ng/g) denote the adsorption amounts at equilibrium and at time, respectively, and k_1_ (/min) and k_2_ (g/ng ⋅ min) denote the rate constants of pseudo-ﬁrst-order and pseudo-second-order adsorption, respectively.

For bilirubin, three milliliters (wet volume) of adsorbents were added to 90 mL of 15 mg/dL bilirubin solution. Then, the mixtures were shaken at 160 rpm for 4 h at 37 °C. Subsequently, 1 mL samples were extracted at a set time and quantitative analysis was performed. The kinetics data were analyzed according to the pseudo-first-order and pseudo-second-order equations:

$$\begin{aligned} ln\left[ \frac{Q_{e}}{Q_{e}-Q_{t}} \right]=k_{1}t\#\left( S5 \right) \end{aligned}$$

$$\begin{aligned} Q_{t}=\frac{k_{2} Q_{e}^{2}t}{1+k_{2}Q_{e}^{2}t}\#\left( S6 \right) \end{aligned}$$

where Qe and Qt (mg/g) denote the adsorption amounts at equilibrium and at time, respectively, and k_1_ (/min) and k_2_ (g/mg⋅min) denote the rate constants of pseudo-ﬁrst-order and pseudo-second-order adsorption, respectively.

**Adsorption isotherms**

Adsorbent materials of varying quality grades (4, 10, 20, 30, 60, 100, and 150 mg) were mixed with 3 mL of 1000 ng/L TNF-α solutions at 160 rpm for 2 h at 37 °C. Next, the adsorption data were further analyzed by the two well-known theoretical models, namely, the Langmuir and Freundlich models[5]:

$$\begin{aligned} \frac{C_{e}}{Q_{e}}=\frac{C_{e}}{Q_{m}}+\frac{1}{K_{L}Q_{m}}\#\left( S7 \right) \end{aligned}$$

$$\begin{aligned} Q_{e}=K_{F}{C_{e}}^{\left( \frac{1}{n} \right)}\#\left( S8 \right) \end{aligned}$$

where C_e_ (ng/L) is the equilibrium concentration, Q_e_ (ng/g) is the adsorption amount, K_L_ and K_F_ (ng/g) denote the equilibrium constants of adsorption, and Q_m_ is the maximum adsorption amount, with the Freundlich adsorption index *n* serving as a gauge of the adsorption strength.

For bilirubin, three milliliters of various bilirubin solution concentrations (2.5, 5, 10, 15, 20, 25, and 30 mg/dL) were mixed with 0.1 mL (wet volume) of adsorbent at 160 rpm for 2 h at 37 °C. Next, the adsorption data were further analyzed by the two well-known theoretical models, namely, the Langmuir and Freundlich models:

$$\begin{aligned} \frac{C_{e}}{Q_{e}}=\frac{C_{e}}{Q_{m}}+\frac{1}{K_{L}Q_{m}}\#\left( S9 \right) \end{aligned}$$

$$\begin{aligned} Q_{e}=K_{F}{C_{e}}^{\left( \frac{1}{n} \right)}\#\left( S10 \right) \end{aligned}$$

where C_e_ (mg/L) is the equilibrium concentration of bilirubin, Q_e_ (mg/g) is the bilirubin adsorption amount, K_L_ and K_F_ (mg/g) denote the equilibrium constants of adsorption, and Q_m_ is the maximum adsorption amount, with the Freundlich adsorption index *n* serving as a gauge of the adsorption strength.

**Effect of microenvironment factors on adsorption**

To investigate the effect of albumin concentration and ion strength, 30 mg of adsorbent was added to 3 mL 1000 ng/L TNF-α solutions with different BSA concentrations (0 - 50 g/L) or NaCl concentrations (0, 0.2, 0.4, 0.6, and 0.8 moL/L) and shaken at 160 rpm for 2 h at 37 °C.

To study the adsorption capacity of TNF-α or bilirubin under different temperatures, 30 mg of adsorbent was added to 3 mL 1000 ng/L TNF-α solutions or 15 mg/dL bilirubin solution with different temperatures (4, 25, 37, and 40 °C) and shaken at 160 rpm for 2 h.

**Adsorption capacity of PSVT/P/EGCG on multiple pro-inflammatory cytokines**

The plasma was spiked with recombinant human cytokines at a target concentration of 1000 ng/L. The adsorbents were added to plasma with a ratio of adsorbent to plasma was 1:10 and shaken at 160 rpm for 2 h at 37°C. The concentrations of different cytokines were determined using Quantikine ELISA kits (R&D Systems, McKinley Place, Minneapolis).

**Cytotoxicity experiment**

The HEK293T cells were cultured in Dulbecco’s Modified Eagle Medium (DMEM) added with 10% fetal bovine serum (FBS) and 1% antibiotics (penicillin-streptomycin) in a 5% CO_2_ humidified incubator at 37°C. The cytotoxicity of PSVT was evaluated using a standard CCK-8 assay. Prior to the experiment, 2 g of the adsorbent was pre-incubated in 10 mL of DMEM for 24 h at 37°C. The supernatant was recovered and then filtering and sterilizing using a 0.22 μm microporous membrane. Cells were seeded in 96-well plates at a density of 1 × 10^3^ cells/well and cultivated for 24 h. After washing with PBS, 200 μL of the supernatant was added to each well and keep incubated at 37°C for 72 h. For the positive control, 100 μL of growth media containing 5% DMSO was employed, while the negative control consisted of 100 μL of DMEM without the adsorbent. Following treatment, the culture media was withdrawn, and 10 μL of CCK-8 reagent and 100 μL of DMEM were applied to each well, followed by incubation at 37°C for 3 h. The optical density (OD) was measured at 450 nm using a multimode plate reader (Synergy 4, USA) to determine cell viability. The relative growth rate (RGR) was calculated using the following formula:

$$\begin{aligned} \mathrm{RGR}\left( \% \right)=\frac{A}{A_{0}}*100\#\left( 2 \right) \end{aligned}$$

where A and A_0_ represent the optical density values of the experimental group and negative control group, respectively.

Furthermore, the following assessment standards were adopted in this study: the cytotoxicity of the positive control group needed to reach at least level 3.

**Supplementary Figures**


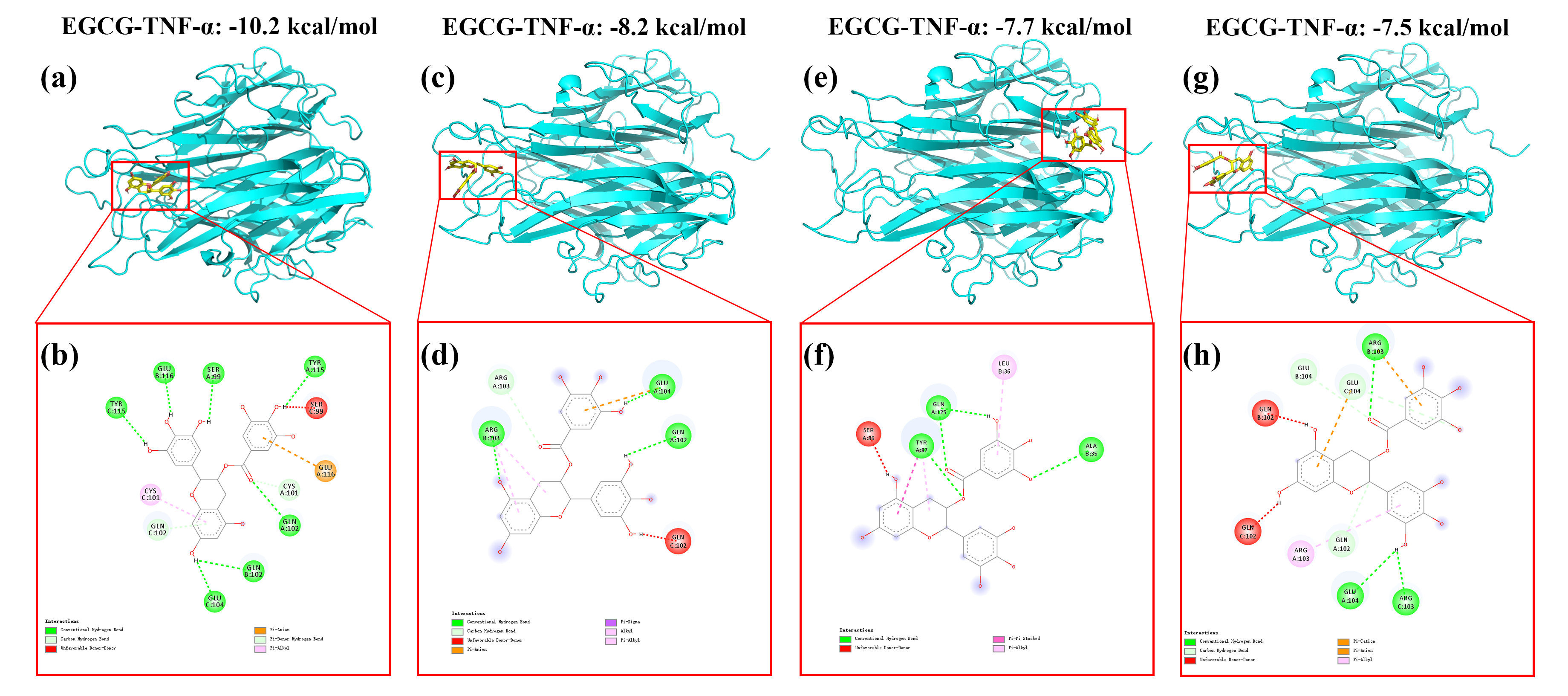


Fig. S1. Docking conformations subjected to cluster analysis, with representative poses from the four lowest binding energy clusters shown.


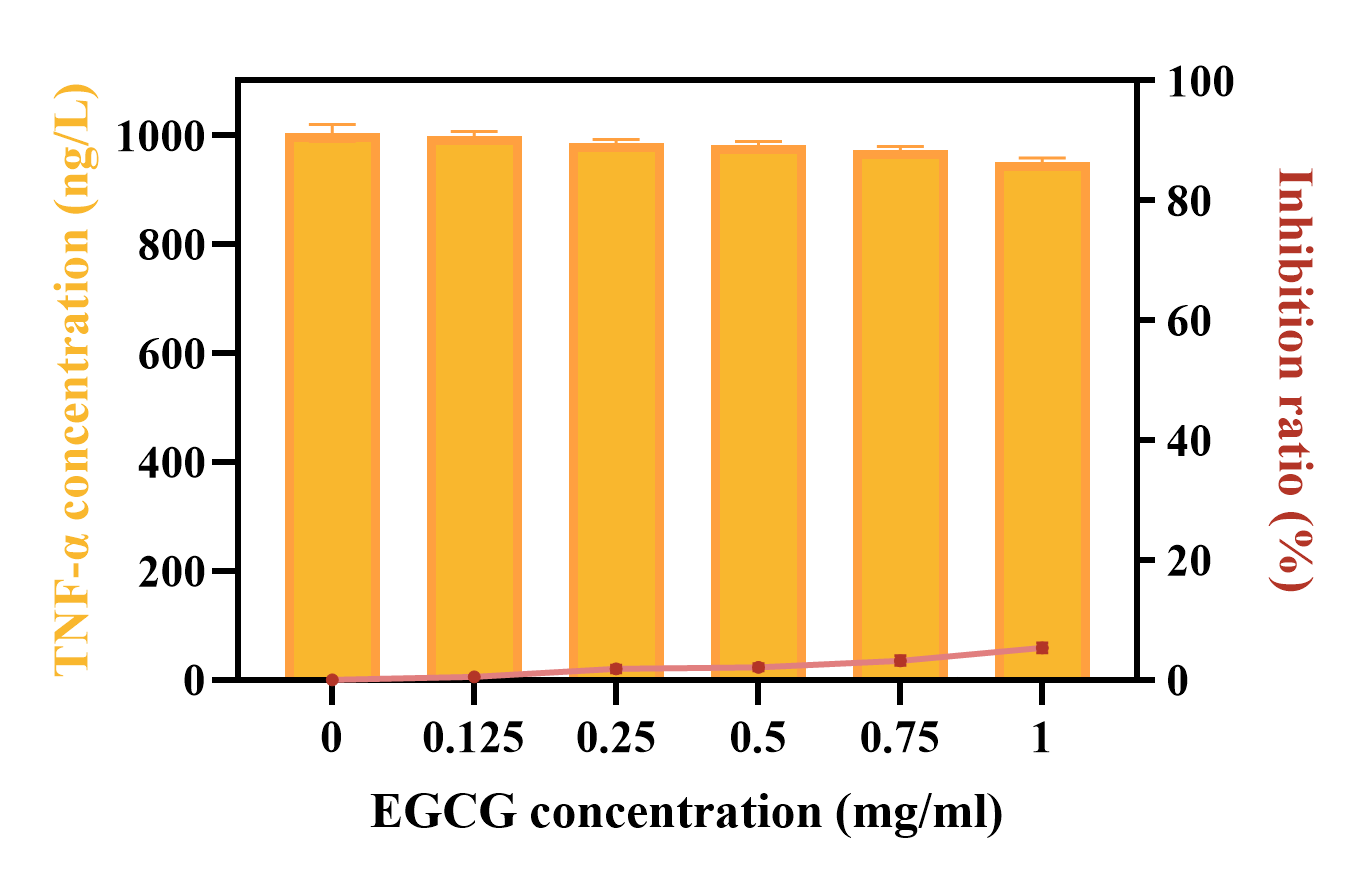


Fig. S2. The interference of EGCG and TNF-α antibody pre-treatment on the detection of TNF-α.

**
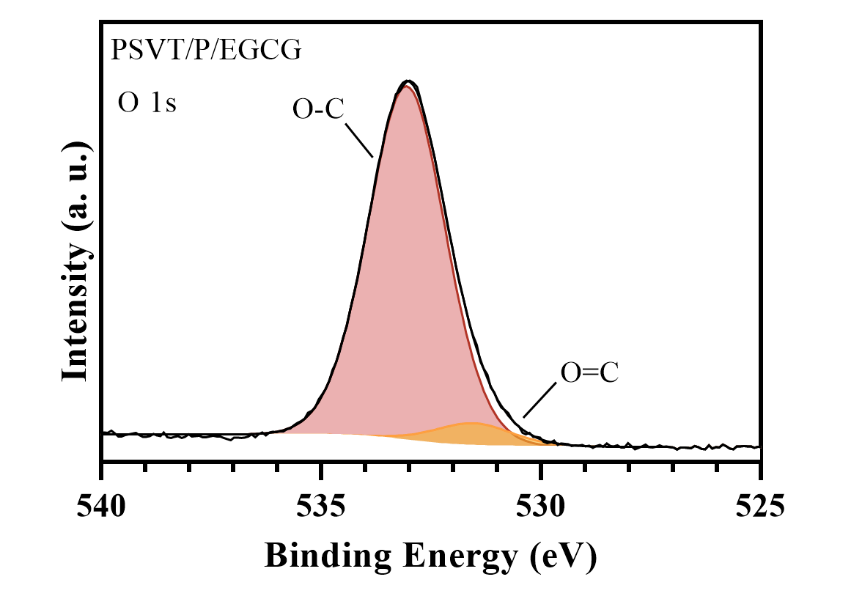
**

Fig. S3. The high-resolution O 1s XPS spectra of PSVT/P/EGCG.

**
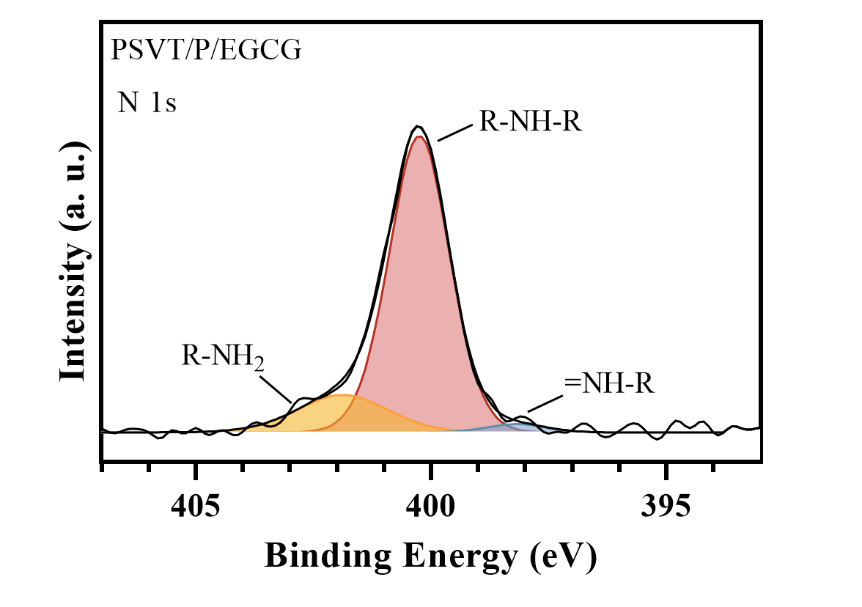
**

Fig. S4. The high-resolution N 1s XPS spectra of PSVT/P/EGCG.

**
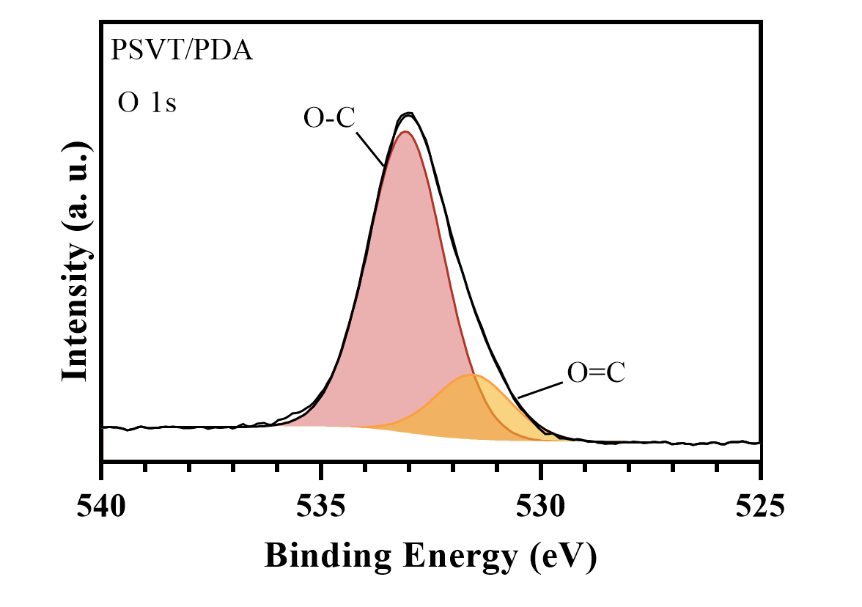
**

Fig. S5. The high-resolution O 1s XPS spectra of PSVT/PDA.

**
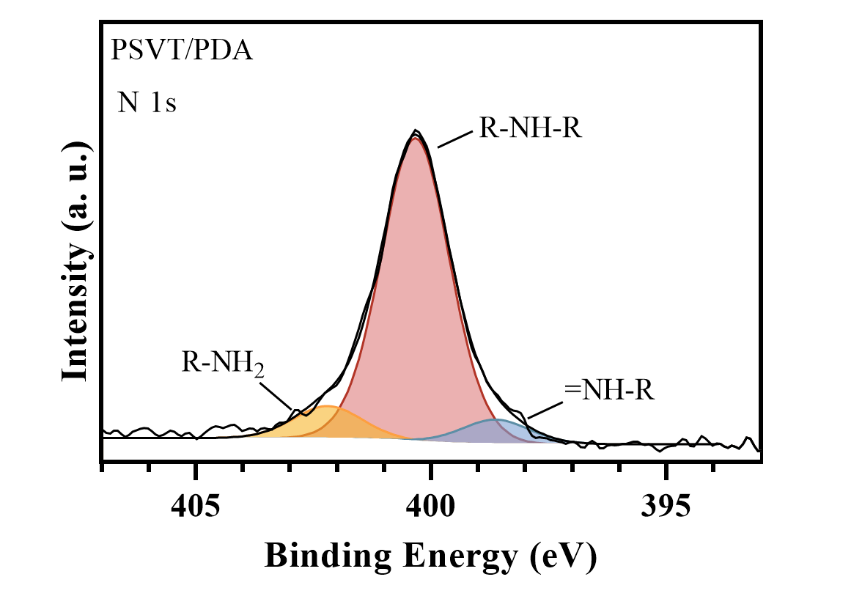
**

Fig. S6. The high-resolution N 1s XPS spectra of PSVT/PDA.


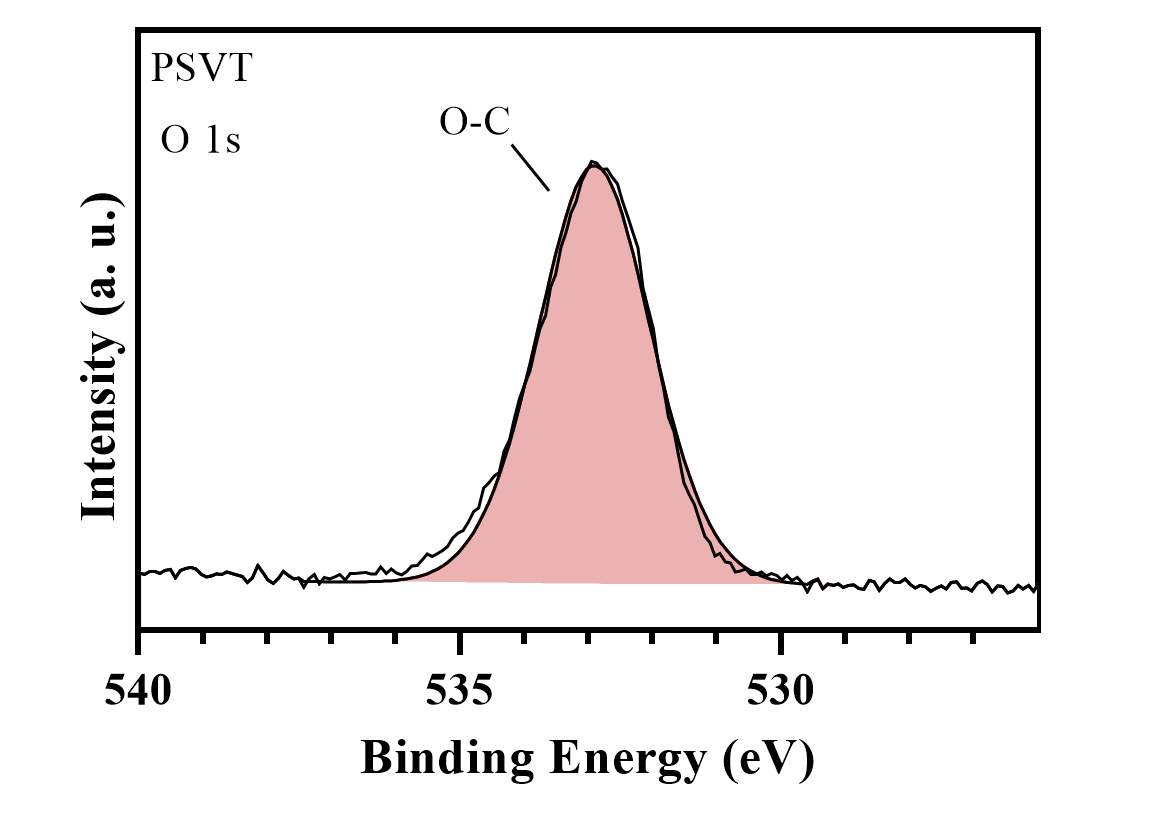


Fig. S7. The high-resolution O 1s XPS spectra of PSVT.


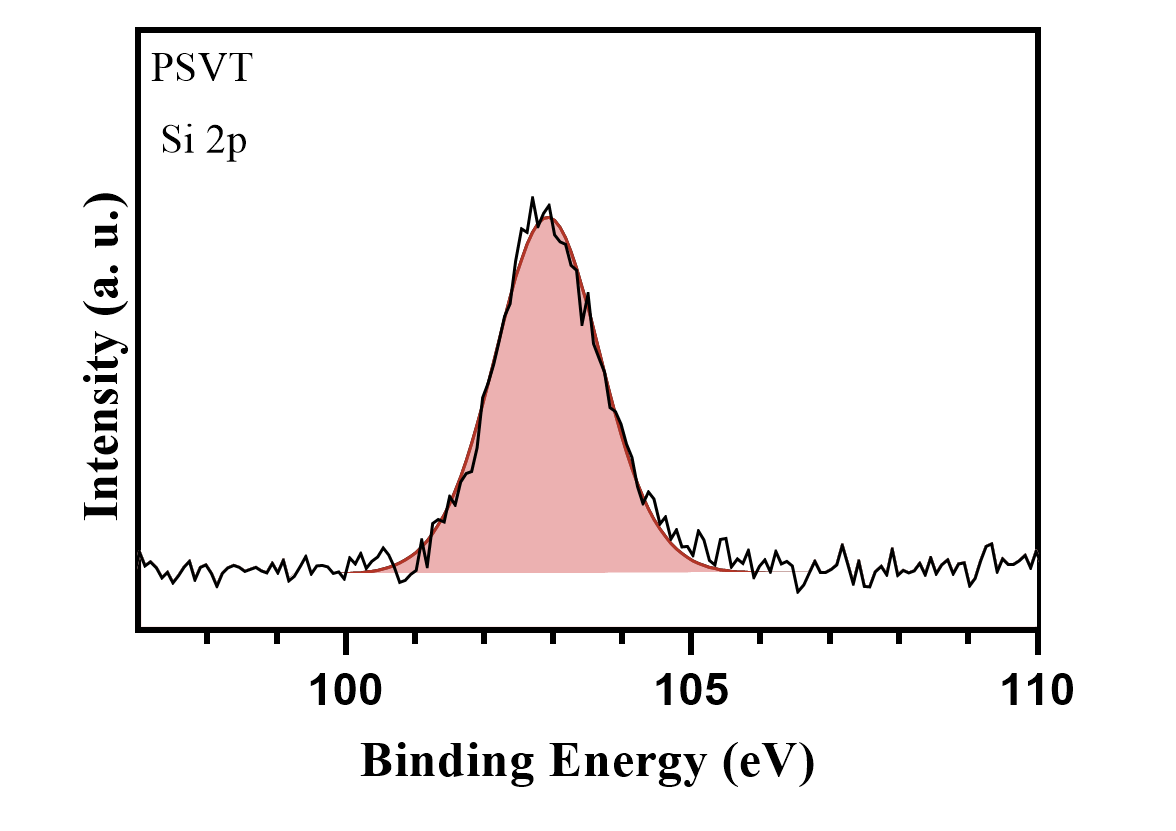


Fig. S8. The high-resolution Si 2p XPS spectra of PSVT.

**
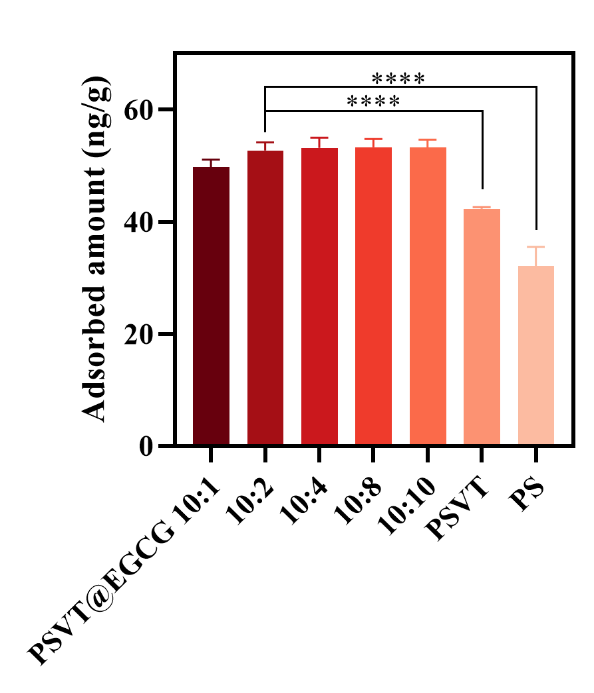
**

Fig. S9. The TNF-α adsorption amount of adsorbents with different EGCG contents.

**
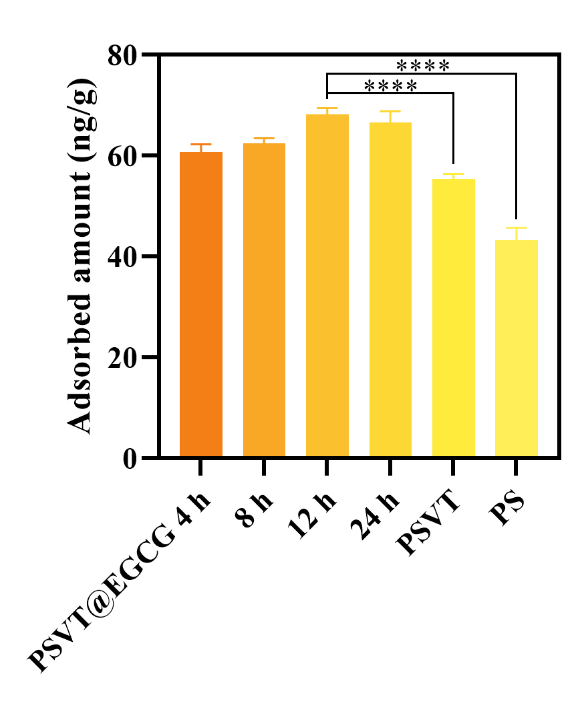
**

Fig. S10. The TNF-α adsorption amount of adsorbents with different EGCG reaction time.


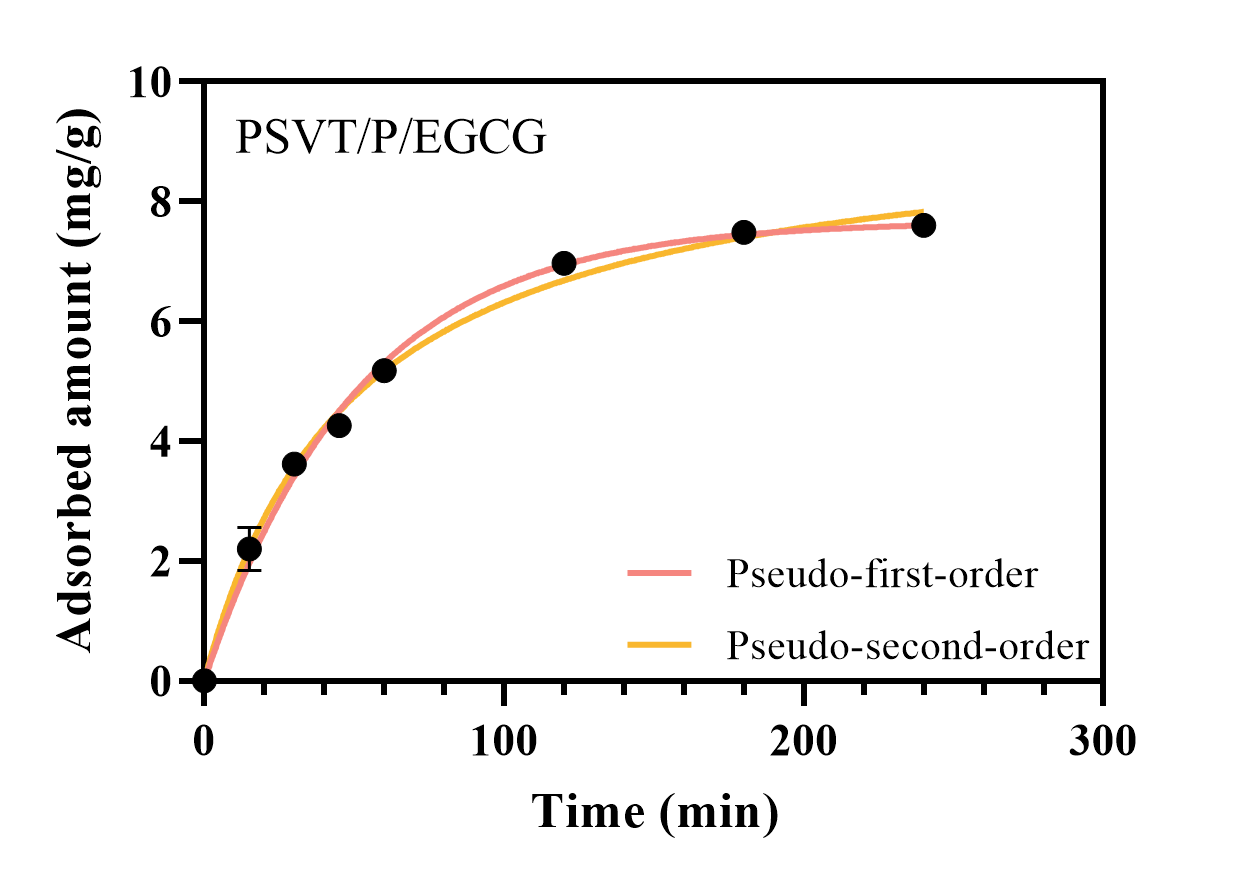


Fig. S11. The bilirubin adsorption kinetic curves of PSVT/P/EGCG.


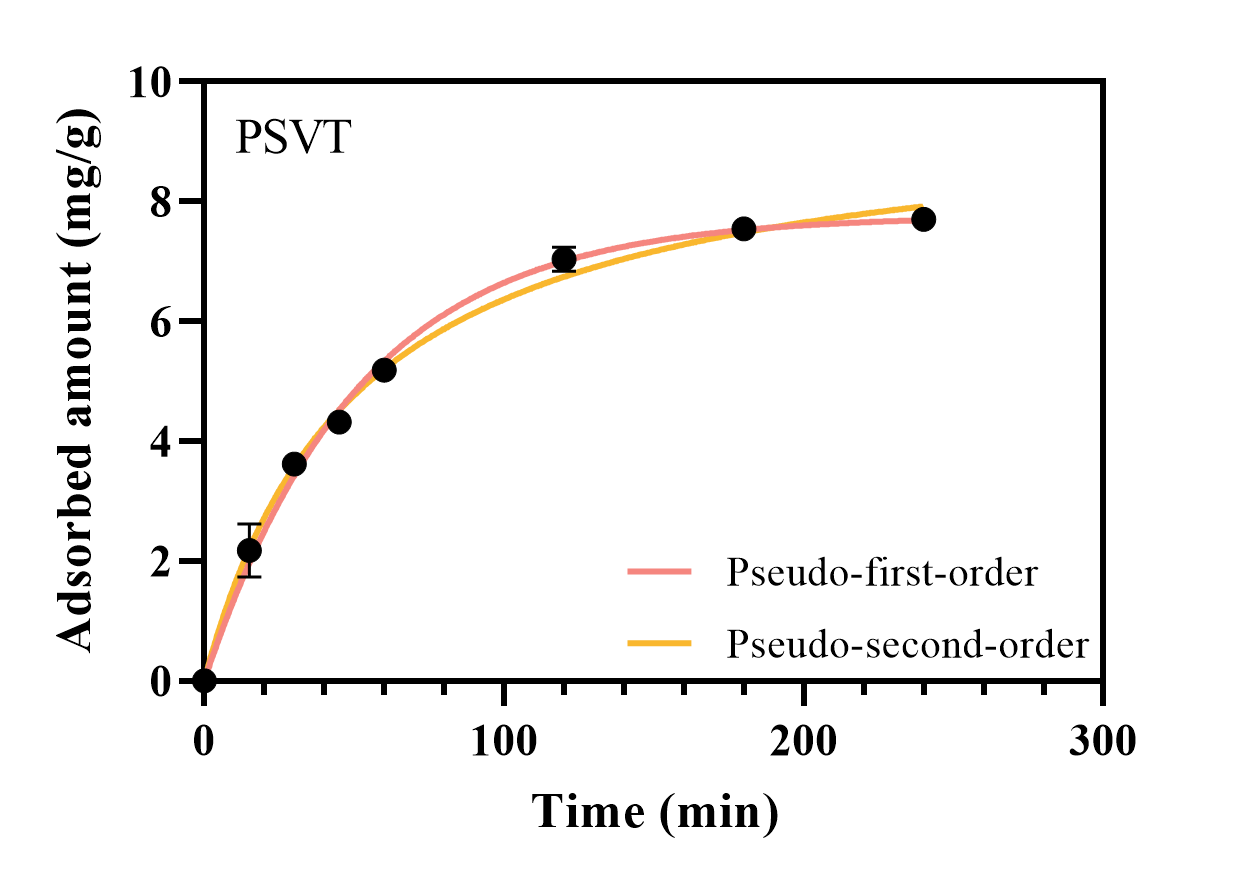


Fig. S12. The bilirubin adsorption kinetic curves of PSVT.


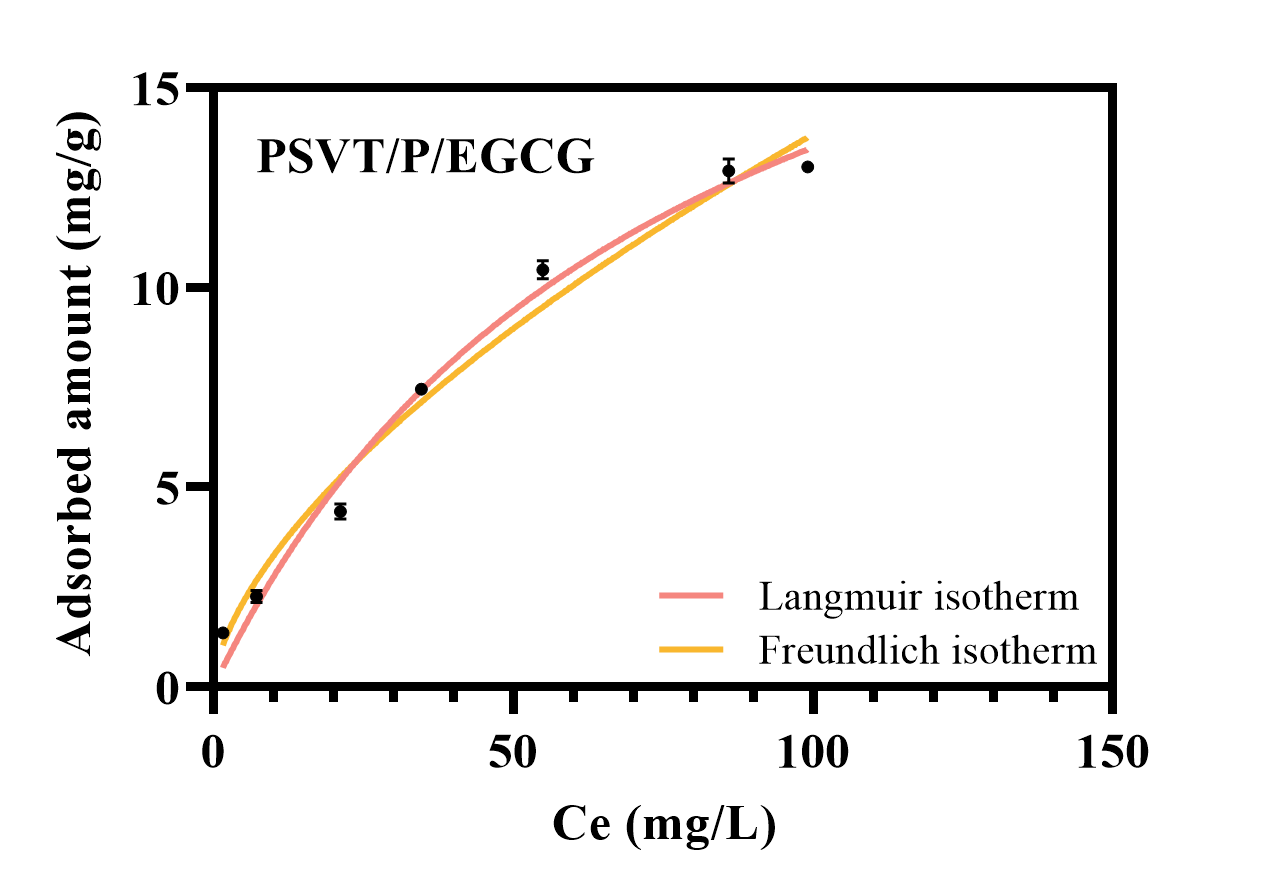


Fig. S13. The bilirubin adsorption isotherm curves of PSVT/P/EGCG.


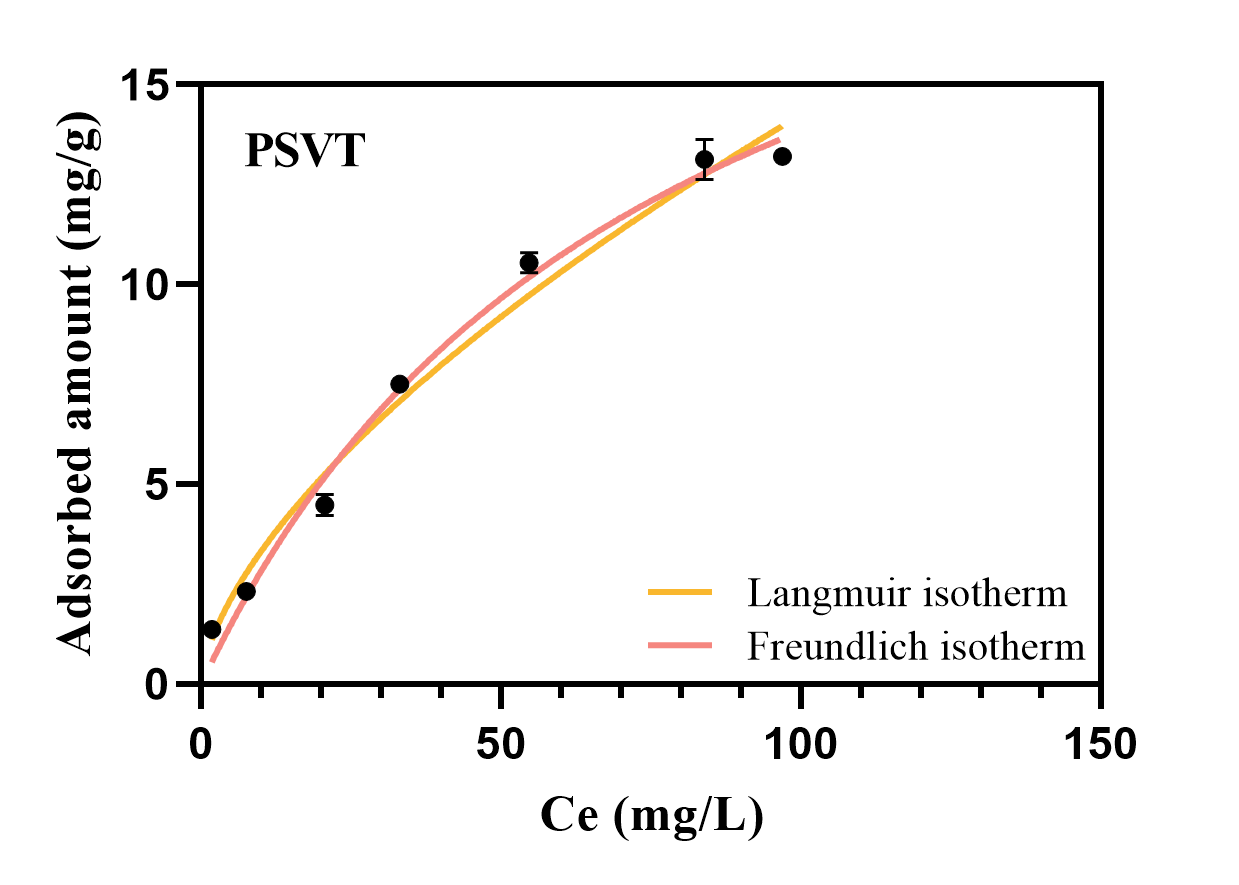


Fig. S14. The bilirubin adsorption isotherm curves of PSVT.


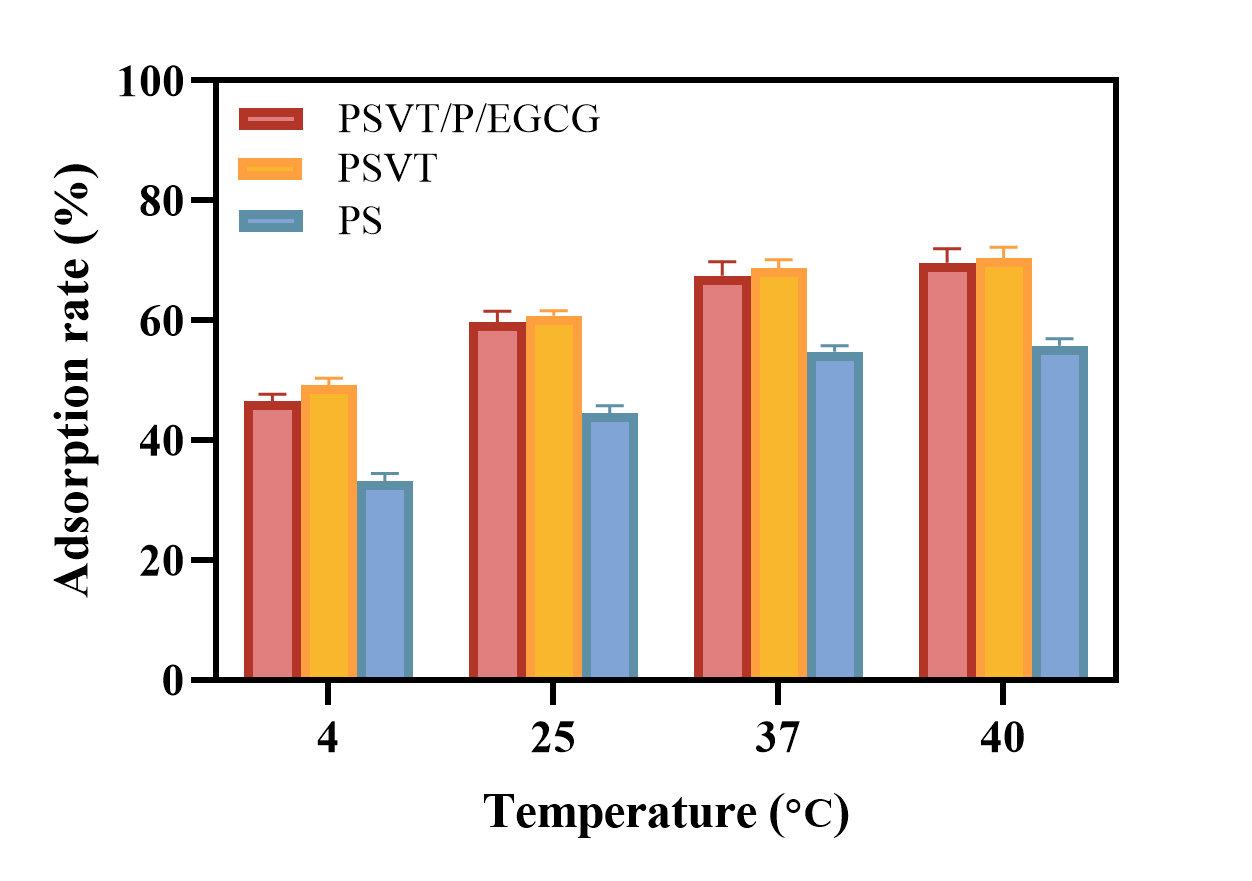


Fig. S15. Effect of temperature on bilirubin adsorption. (C_BSA_ = 30 g/L, t = 2 h).


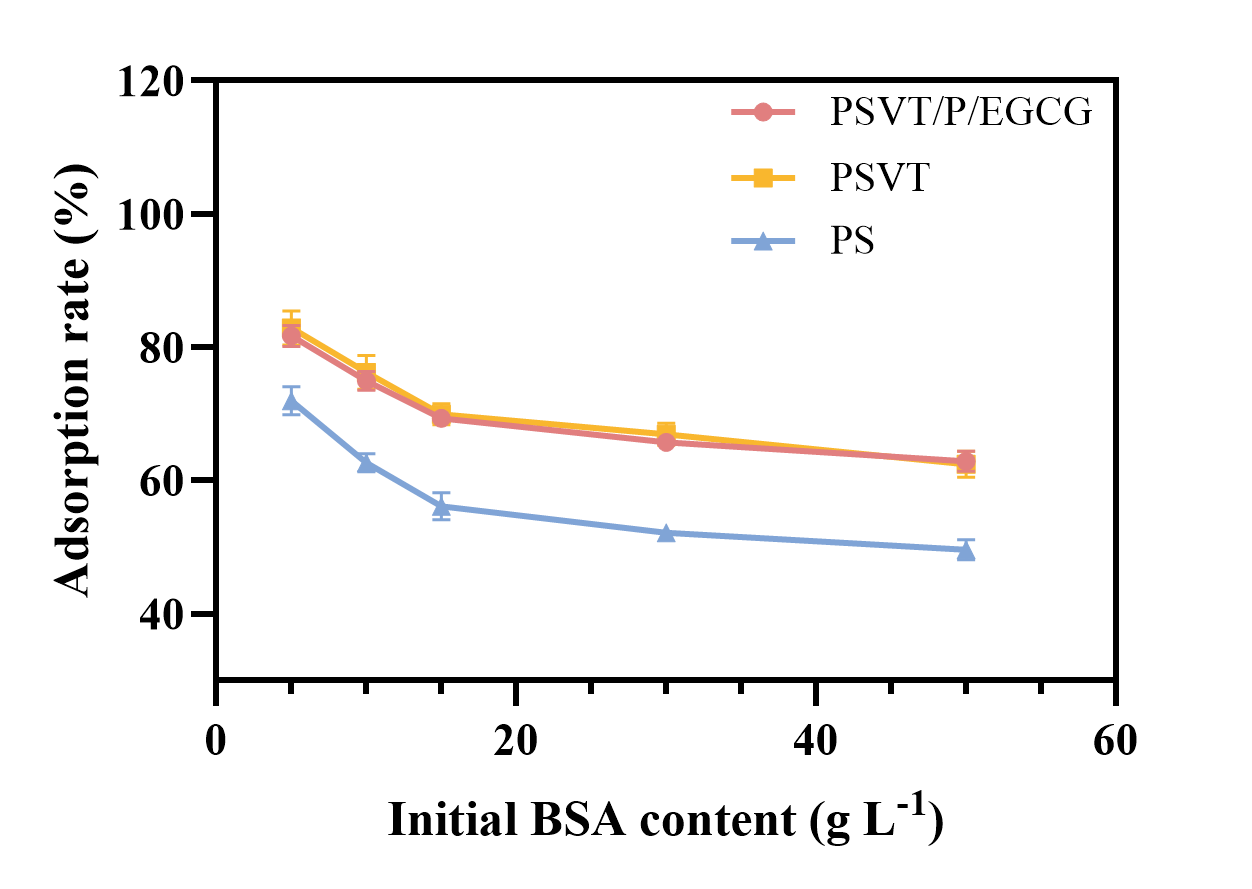


Fig. S16. Effect of albumin content on bilirubin adsorption. (T = 37°C, t = 2 h).

**Supplementary Tables**

Table. S1. The particle size distribution and Gaussian fitting for three adsorbents.

| **Parameter** | **PSVT/P/EGCG** | **PSVT/PDA** | **PSVT** |
| --- | --- | --- | --- |
| A (%*μm) | 17.72 | 16.79 | 18.71 |
| μ (μm) | 464.7 | 460.5 | 445.3 |
| σ (μm) | 124.1 | 128.3 | 113.6 |
| D_90_ (μm) | 688 | 697 | 648 |
| D_50_ (μm) | 467 | 461 | 450 |
| D_10_ (μm) | 325 | 315 | 319 |
| Particle size (μm) | 452 | 445 | 437 |

Table. S2. Physical parameters of PSVT/P/EGCG, PSVT/PDA, and PSVT.

| **Adsorbents** | **S_BET_ (cm^2^/g)** | **T_p_ (cm^3^/g)** | **D_p_ (nm)** |
| --- | --- | --- | --- |
| **PSVT/P/EGCG** | 484.87 | 0.6970 | 5.7563 |
| **PSVT/PDA** | 575.96 | 0.7754 | 5.3849 |
| **PSVT** | 777.12 | 1.1807 | 6.0783 |

Table. S3. The elemental content of three adsorbents detected by XPS.

| **Adsorbents** | **C** | **N** | **O** |
| --- | --- | --- | --- |
| **PSVT/P/EGCG** | 84.78 | 1.30 | 13.93 |
| **PSVT/PDA** | 85.84 | 2.43 | 11.73 |
| **PSVT** | 92.26 | 0 | 7.74 |

Table. S4. Three adsorbents’ adsorption kinetic for TNF-α adsorption.

| **Adsorbents** | ***Q*_e,exp_**  **(ng/g )** | **Pseudo-first-order** | | **Pseudo-second-order** | |
| --- | --- | --- | --- | --- | --- |
|  |  | ***Q*_e,calc,1_**  **(ng/g )** | ***R*_1_^2^** | ***Q*_e,calc,2_**  **(ng/g )** | ***R*_2_^2^** |
| **PSVT/P/EGCG** | 102.45 | 88.29 | 0.9278 | 103.01 | 0.9676 |
| **PSVT** | 72.97 | 71.22 | 0.9806 | 91.11 | 0.9797 |
| **PS** | 60.28 | 64.90 | 0.9884 | 92.56 | 0.9883 |

Table. S5. Two adsorbents’ adsorption kinetic for bilirubin adsorption.

| **Adsorbents** | ***Q*_e,exp_**  **(mg/g )** | **Pseudo-first-order** | | **Pseudo-second-order** | |
| --- | --- | --- | --- | --- | --- |
|  |  | ***Q*_e,calc,1_**  **(mg/g )** | ***R*_1_^2^** | ***Q*_e,calc,2_**  **(mg/g )** | ***R*_2_^2^** |
| **PSVT/P/EGCG** | 7.63 | 7.669 | 0.9943 | 9.440 | 0.9940 |
| **PSVT** | 7.75 | 7.763 | 0.9940 | 9.584 | 0.9936 |

Table. S6. Three adsorbents’ isothermal constants for TNF-α adsorption.

| **Adsorbents** | **Langmuir model** | | | **Freundlich model** | | |
| --- | --- | --- | --- | --- | --- | --- |
|  | **Qm (ng/g)** | **K_L_** | **R^2^** | **K_F_** | **1/n** | **R^2^** |
| **PSVT/P/EGCG** | 263.6 | 0.003138 | 0.9542 | 5.404 | 0.5453 | 0.9880 |
| **PSVT** | 77.01 | 0.005556 | 0.9121 | 3.589 | 0.4398 | 0.9623 |
| **PS** | 45.32 | 0.008653 | 0.9524 | 4.464 | 0.3355 | 0.9530 |

Table. S7. Two adsorbents’ isothermal constants for bilirubin adsorption.

| **Adsorbents** | **Langmuir model** | | | **Freundlich model** | | |
| --- | --- | --- | --- | --- | --- | --- |
|  | **Qm (mg/g)** | **K_L_** | **R^2^** | **K_F_** | **1/n** | **R^2^** |
| **PSVT/P/EGCG** | 23.91 | 0.01299 | 0.9857 | 0.7767 | 0.6253 | 0.9809 |
| **PSVT** | 24.43 | 0.01306 | 0.9868 | 0.7744 | 0.6325 | 0.9815 |

Table. S8. Bilirubin adsorption of adsorbents from hyperbilirubinemic plasma.

| **Adsorbents** | **Direct Bilirubin** | | **Indirect Bilirubin** | | **Total Bilirubin** | |
| --- | --- | --- | --- | --- | --- | --- |
|  | **Concentration (mg/dL)** | **Adsorption rate (%)** | **Concentration (mg/dL)** | **Adsorption rate (%)** | **Concentration (mg/dL)** | **Adsorption rate (%)** |
| **PSVT/P/EGCG** | 1.11±0.07 | 56.81±2.89 | 0.74±0.02 | 67.18±0.83 | 1.85±0.06 | 61.64±1.16 |
| **PSVT** | 1.08±0.04 | 58.02±1.46 | 0.66±0.03 | 70.71±1.46 | 1.74±0.02 | 63.93±0.50 |
| **PS** | 1.58±0.17 | 38.47±6.76 | 1.18±0.02 | 47.29±0.85 | 2.77±0.15 | 42.58±3.21 |
| **CYT** | 2.07±0.11 | 19.55±4.20 | 1.63±0.01 | 27.25±0.32 | 3.70±0.11 | 23.14±2.33 |
| **BPR** | 1.21±0.07 | 53.02±2.59 | 0.64±0.03 | 71.67±1.40 | 1.85±0.04 | 61.71±0.74 |
| **Stock Solution** | 2.57±0.07 | NA | 2.25±0.02 | NA | 4.82±0.06 | NA |

Table. S9. Bilirubin adsorption under dynamic perfusion conditions.

| **Adsorbent** | **Direct Bilirubin** | | **Indirect Bilirubin** | | **Total Bilirubin** | |
| --- | --- | --- | --- | --- | --- | --- |
|  | **Concentration (mg/dL)** | **Adsorption rate (%)** | **Concentration (mg/dL)** | **Adsorption rate (%)** | **Concentration (mg/dL)** | **Adsorption rate (%)** |
| **PSVT/P/EGCG** | 1.20±0.04 | 53.32±1.60 | 0.79±0.03 | 64.87±1.25 | 1.99±0.02 | 58.70±0.48 |
| **Stock Solution** | 2.57±0.07 | NA | 2.25±0.02 | NA | 4.82±0.06 | NA |

Table. S10. The Hemolysis rate of adsorbents.

| **Adsorbents** | **Hemolysis rate (%)** |
| --- | --- |
| **PSVT/P/EGCG** | 1.16 |
| **PSVT** | 0.14 |
| **PS** | 0.20 |
| **CYT** | 0.14 |
| **BPR** | 19.54 |

Table. S11. The blood biochemical assay results of adsorbents.

| **Adsorbents** | **PSVT/P/EGCG** | **PSVT** | **PS** | **CYT** | **BPR** |
| --- | --- | --- | --- | --- | --- |
| ALT (U/L) | 28 | 27 | 28 | 28 | 27 |
| AST (U/L) | 34 | 35 | 34 | 33 | 35 |
| ALP (U/L) | 76.2 | 77.3 | 76.3 | 75.4 | 75.5 |
| GGT (U/L) | 33.9 | 34.7 | 34.6 | 34.0 | 34.3 |
| LDH (U/L) | 171 | 165 | 163 | 152 | 148 |
| UA (μmol/L) | 301.1 | 303.4 | 297.7 | 302.5 | 300.7 |
| Urea (mmol/L) | 4.17 | 4.23 | 4.06 | 4.15 | 4.07 |
| CREA (μmol/L) | 52.7 | 58.1 | 56.3 | 55.4 | 54.6 |
| Ca (mmol/L) | 2.05 | 2.05 | 2.08 | 2.06 | 2.03 |
| K (mmol/L) | 3.87 | 3.86 | 3.81 | 3.84 | 3.85 |
| Na (mmol/L) | 143.7 | 144.3 | 144.1 | 143.7 | 144.1 |
| Cl (mmol/L) | 105.9 | 106.4 | 105.8 | 106.2 | 106.1 |

**References**

1. Fernández J, Lozano M, Torres M, Horrillo R, Afonso N, Núñez L, Mestre A, Pérez A, Cid J, Costa MJJR. Effect of plasma exchange with albumin replacement on albumin functionality and organ dysfunction in acute-on-chronic liver failure. 2024;6:101017.

2. Doumas BT, Kwok-Cheung PP, Perry BW, Jendrzejczak B, McComb RB, Schaffer R, Hause LLJCc. Candidate reference method for determination of total bilirubin in serum: development and validation. 1985;31:1779-1789.

3. Chai Y, Liu Z, Du Y, Wang L, Lu J, Zhang Q, Han W, Wang T, Yu Y, Sun L, Ou L. Hydroxyapatite reinforced inorganic-organic hybrid nanocomposite as high-performance adsorbents for bilirubin removal in vitro and in pig models. *Bioactive Materials* 2021;6:4772-4785.

4. Liuyang X, Yang H, Huang S, Zhang Y, Xia S. Resource utilization of secondary pyrolysis oil-based drilling cuttings ash for removing Cr (VI) contaminants: Adsorption properties, kinetics and mechanism. *Journal of Environmental Chemical Engineering* 2020;8:104474.

5. Bashir M, Tyagi S, Annachhatre AP. Adsorption of copper from aqueous solution onto agricultural Adsorbents: Kinetics and isotherm studies. *Materials Today: Proceedings* 2020;28:1833-1840.
